# Reference Gene Selection for Quantitative Real-Time PCR Normalization in *Reaumuria soongorica*

**DOI:** 10.1371/journal.pone.0104124

**Published:** 2014-08-12

**Authors:** Xia Yan, Xicun Dong, Wen Zhang, Hengxia Yin, Honglang Xiao, Peng Chen, Xiao-Fei Ma

**Affiliations:** 1 Key Laboratory of Inland River Ecohydrology, Cold and Arid Regions Environmental and Engineering Research Institute, Chinese Academy of Sciences, Lanzhou, P. R. China; 2 Key Laboratory of Stress Physiology and Ecology in Cold and Arid Regions of Gansu Province, Cold and Arid Regions Environmental and Engineering Research Institute, Chinese Academy of Sciences, Lanzhou, P. R. China; 3 Department of Radiobiology, Institute of Modern Physics, Chinese Academy of Sciences, Lanzhou, P. R. China; 4 Faculty of Plant Science and Technology, Huazhong Agricultural University, Wuhan, P. R. China; National Institute of Genomic Medicine, Mexico

## Abstract

Despite its superiority for evaluating gene expression, real-time quantitative polymerase chain reaction (qPCR) results can be significantly biased by the use of inappropriate reference genes under different experimental conditions. *Reaumuria soongorica* is a dominant species of desert ecosystems in arid central Asia. Given the increasing interest in ecological engineering and potential genetic resources for arid agronomy, it is important to analyze gene function. However, systematic evaluation of stable reference genes should be performed prior to such analyses. In this study, the stabilities of 10 candidate reference genes were analyzed under 4 kinds of abiotic stresses (drought, salt, dark, and heat) within 4 accessions (HG010, HG020, XGG030, and XGG040) from 2 different habitats using 3 algorithms (geNorm, NormFinder, and BestKeeper). After validation of the ribulose-1,5-bisphosphate carboxylase/oxygenase large unite (*rbcL*) expression pattern, our data suggested that histone H2A (*H2A*) and eukaryotic initiation factor 4A-2 (*EIF4A2*) were the most stable reference genes, cyclophilin (*CYCL*) was moderate, and elongation factor 1α (*EF1α*) was the worst choice. This first systematic analysis for stably expressed genes will facilitate future functional analyses and deep mining of genetic resources in *R. soongorica* and other species of the *Reaumuria* genus.

## Introduction

Gene expression analysis is increasingly important in many fields of biological research. Understanding gene expression patterns is expected to provide insight into complex regulatory networks and will help to identify which genes are relevant to new biological processes. Recently developed methods to measure transcript abundance have been frequently applied in many model biological systems; however, these approaches require the same normalization procedures as traditional mRNA quantification methods. Real-time quantitative polymerase chain reaction (qPCR) is one of the most common technologies used for gene expression and transcriptome analysis and is characterized by high sensitivity and specificity, good reproducibility, a widely dynamic quantification range, and high-throughput capacity for a limited number of target genes [1]–[3]. Despite its superiority over other methods available for evaluating gene expression, qPCR remains underused due in part to conflicting results and weak repeatability. Traditional reference genes, such as actin (*ACT*), ubiquitin (*UBQ*), alpha-tubulin (*TUA*), eukaryotic initiation factor 4A-2 (*EIF4A2*), 60S ribosomal protein L2 (*L2*), TIP41-like protein (*TIP41*), elongation factor 1-alpha (*EF1α*), cyclophilin (*CYCL*), histone H2A (*H2A*) and DNAJ-like protein (*DNAJ*), are mostly used in model plants and crops [4]–[6] and do not always maintain stable expression levels among different tissues, experimental conditions [7]–[12], or species [7], [11]–[16]. Systematic validations of reference genes have mainly focused on models and important crop species, such as Arabidopsis [17], rice [6], poplar [18], soybean [19], wheat [14], barley [20], tomato [21], grape [22], and potato [5]. However, with the increasing importance of agronomy and ecology, greater numbers of non-model plants are being assessed with molecular functional analyses, and it is crucial to identify proper reference genes for new species. To date, reference gene selection has been performed for some non-model plants like bamboo [23], peach [15], *Caragana intermedia* [12], and eggplant [24], but seldom in desert plants.

*Reaumuria* (Tamaricaceae) plants are perennial xeric shrubs widely distributed in arid regions in North Africa, Asia, and southern Europe. *R. soongorica* (2n = 22, 778 Mb genome size) [25] is a constructive and dominant species of desert ecosystems in central Asia [26]. It has evolved typical phenotypes of desert plants, such as an extremely thick cuticle; hollow stomata; specialized leaf shape; and a root system that reduces the transpiration rate, increases water use efficiency, and maintains stem vigor to survive desiccation [27], [28]. Thus, *R. soongorica* is a good species to study the molecular mechanisms of drought adaptation. Many studies have attempted to elucidate the mechanisms of the adaptation and tolerance of *R. soongorica* under the extreme drought conditions [29]–[33], which suggested its more and more importance of ecology. It is in urgent to systematically identify appropriate references genes for *R. soongorica*, for better understanding of the processes and molecular functions of its potential gene resources since its transcriptome analysis had been released [34].

Three statistical algorithms packages have been developed to assess reference gene stability: geNorm [35], NormFinder [11], and BestKeeper [36]. In this study, the stability of 10 candidate reference genes used in many plants (*ACT*, *UBQ*, *TIP41*, *CYCL*, *EF1α*, *EIF4A*, *H2A*, *L2*, *DNAJ*, and *TUA*) were assessed based on their expression abundance in two contrasting populations under four stress conditions. To validate the best candidate reference genes, the expression levels of the ribulose-1,5-bisphosphate carboxylase/oxygenase (*rbcL*) homolog were analyzed in comparison to 10 reference genes. This work will facilitate future studies on *R. soongorica* gene expression, speed up functional analyses of special genetic resources, and improve our understanding of the molecular mechanisms of drought adaptation.

## Materials and Methods

### Ethics Statement

*R. soongorica* is a species widely distributed in Shashichang, Jingtai County, Gansu province and other arid regions, and it is not included on any list of endangered or protected species. Before collecting the samples, oral permission was obtained from the local management of forestry after sending introduction letters from the CAREERI (Cold and Arid Regions Environmental and Engineering Research Institute, Chinese Academy of Sciences).

### Plant Materials and Treatments

Seeds of four accessions (HG010, HG020, XGG030, and XGG040) were collected from two different habitats (Honggu [HG] N36°15′20.16″, E103°1′29.82″, annual precipitation [AP] ∼400 mm based on WorldClim database 1950–2000; Xiaogangou [XGG] N36°54′21.60″, E103°53′31.44″, AP ∼150 mm) were sampled in the year 2011. After 2 years in frozen storage at −20°C to completely break physiological dormancy, the seeds were surface sterilized with 0.1% sodium hypochlorite and rinsed with distilled water. The sterilized seeds were then grown in MS-medium with photosynthetically active radiation under long day condition (16/8-hour light-dark cycles); the relative humidity was 75%, and the temperatures were 25°C in the light and 22°C in the dark. After 2 weeks, 10 seedlings from each accession were collected for RNA extraction. For drought stress, seedlings were subjected to 15% PEG (polyethylene glycol) for 1 hour. Heat stress treatment was performed at 38°C. For salt stress, plants were treated with 100 mM NaCl for 3 hours. For dark stress, plants were wrapped with foil paper for 2 hours. Control plants were kept at 25°C during light hours on MS-medium.

### Total RNA Isolation and cDNA Synthesis

Total RNA was extracted using an E.Z.N.A. plant RNA kit (Omega Bio-tek), and the remaining DNA was removed by RNase-free DNase (Omega Bio-tek) according to the instruction manual. Total RNA concentration and purity were determined with ratio optical density (OD) 260/280 by NanoDrop1000 (Thermo Scientific). All the samples passed QC with the ratio OD260/280 between 1.9 and 2.2 and ratio OD260/230 <2.0. RNA integrity was verified by 1.5% agarose gel electrophoresis with two clear bands of 28S/18S ribosomal RNA. In each treatment and control, three biological replicates of RNA samples were extracted from at least 10 seedlings of each genotype first, and then mixed equally as genotype representative sample. For each representative sample, 1 µg total RNA was reverse transcribed in a 20-µl volume reaction with oligo dT primers by the RevertAidTM First Strand cDNA Synthesis Kit (Fermentas). In total, we got 20 cDNA samples (four genotypes with four treatments and one control) to be analyzed in this study. The cDNAs were diluted 1∶10 with nuclease-free water prior to the qPCR analyses.

### Selection and Validation of Candidate Genes

Candidate reference genes were screened based on the transcriptome *de novo* assembly sequences [34] with lengths >1 kb and transcription abundance FPKM (fragments per kilobase of exon per million fragments mapped) values >5. Unigenes were annotated by National Center for Biotechnology Information (NCBI) non-redundant protein (Nr) database with E-values less than 1e^−28^. A reciprocal BLAST hits approach was then conducted against Arabidopsis with a permissive E-value cutoff of 10^−3^ to obtain hits to confirm the gene homologs in *R. soongorica*. The final list of 10 candidate reference genes included *ACT*, *EF1α*, *TUA*, *TIP41*, *DNAJ*, *UBQ*, *CYCL*, *EIF4A2*, *H2A*, and *L2*, which were assessed in later expression analyses (Table 1).

**Table 1:** Candidate reference gene descriptions.

Gene Symbol	Length (bp)	Tm (°C)	LinRegPCR Efficiency	5′-Sequences	3′-Sequences	GeneID	Gene Length	Nr-Annotation
*ACT*	145	80.57	1.834	AGCAAGGTCAAGACGAAGGA	TGTTGCTATTCAGGCTGTGC	CL448.Contig2_A	1784	Actin
*CYCL*	287	83.56	1.946	CCCTGAAGCATGAAGCTAGG	GCCTTGATCTGGTGTTTGGT	Unigene10547_A	1193	Cyclophilin
*DNAJ*	141	81.91	1.911	GATTATCTCAGCGCGCCTAC	GATTGGTTTCGGGTCTTTCA	Unigene46962_A	819	DnaJ-like protein
*EF1α*	128	81.88	1.929	CCCCAGGTTAATCCTTCCAT	GGAAAGAAGCCCAAGGAATC	Unigene18820_A	1127	elongation factor 1 alpha
*EIF4A2*	116	77.24	1.854	TCAGCAACATTTGCAGGAAG	TTTGGAAGAAAGGGTGTTGC	CL9396.Contig2_A	1717	Eukaryotic initiation factor 4A-2
*H2A*	164	81.54	1.875	GGCGCTGCAAAGAAGTCTAC	AGGACCTCAGCAGCAAGGTA	CL9675.Contig2_A	818	Histone H2A
*L2*	280	82.9	1.885	TCATTGCGAACGAAACTCTG	ACGGTGACAATTCAGGGAAG	CL8967.Contig2_A	1342	60S ribosomal protein L2
*TIP41*	110	79.16	1.899	GGTTTCTGCTCTTGCGTTTC	CCGTTTAATGATGGGGAATG	CL7670.Contig1_A	1134	TIP41-like protein
*TUA*	264	82.82	1.893	CAACAAGCCGCTAAGGAAAG	GCCTTCGTCCACTGGTATGT	CL12940.Contig1_A	1916	alpha-tubulin
*UBQ*	113	80.16	1.878	CCGGCCTTCTGGTAAACATA	TTGAGGAAGGAATGCCCTAA	CL5323.Contig1_A	471	Ubiquitin
*rbcL* *	185	80.93	1.890	AGAGCACGTAGGGCTTTGAA	GACAACTGTGTGGACCGATG	Unigene19142_A	5856	Ribulose-1,5-bisphosphate carboxylase oxygenase

Notes:

* used for normalization validation under stress conditions in four accessions.

*rbcL* gene (ribulose-1,5-bisphosphate carboxylase/oxygenase large subunit, chloroplast) was chosen for the validation of reference genes. We tookUnigene19142_A, a *rbcL* homolog in the *R. soongorica de novo* transcriptome sequences (from Shi et.al. 2013) to predict its peptide sequence and gene structure with the GENSCAN Web Server at MIT (http://genes.mit.edu/GENSCAN.html). The protein sequence was then blasted against Nr database in NCBI and got the best hit ribulose-1,5-bisphosphate carboxylase/oxygenase large subunit (chloroplast) in *Eucalyptus umbra* (YP_008575886.1, with total score of 978, coverage of 100% and identity of 99%). The protein sequences of the *rbcL* homolog was well aligned with those from other species in File S3.pdf, which suggested the high homologous of *rbcL* genes to each other. The annotation information of the ten reference genes and one validation gene were listed in the Table S1.

### qPCR Primer Design and Verification of Amplification

Primers of each reference gene were designed by the platform OligoPerfect Designer (Invitrogen), with optimal melting temperatures 60°C, optimal GC content 50%, optimal primer size 20 bp, and product size was between 100 and 300 bp (Table 1, and Figure S1). Each primer showed good specificity and efficiency (File S1 and S2). PCR efficiencies per amplicon were calculated by LinRegPCR [37]–[39], and the mean PCR efficiency between 1.8 and 2.0 from three technology replicates was regarded as the efficiency of one sample. The final primer efficiency was obtained from the average of all sample amplified with each pair of primer.

qPCR amplification products of the 10 candidate reference from one genotype (XGG030) were sequenced with ABi 3730 automated DNA Sequencer. Both the product sizes and sequences of amplificiaton product were consistent with the unigenes from *de novo* sequencing (File S4).

### qPCR

qPCR experiments were conducted in the Stratagene Mx3000P QPCR system (Agilent Technologies), in which combined gene amplification, detection and data analysis steps with FAM, HEX, ROX, and TET filters. A 20-µl reaction system volume was used for amplification. Each reaction contained 10 µl DyNAmo Flash SYBR Green qPCR Kit Master Mix (Thermo Scientific), 0.5 µl each of forward and reverse primer, 0.2 µl F-402 buffer, 2 µl cDNA synthesized from total RNA. The PCR program contained an initial denaturation step of 5 min at 95°C, followed by denaturation for 15 s at 95°C, annealing for 30 s at 60°C, and extension for 30 s at 72°C for 40 cycles. Three technical replicates were used in qPCR experiments, and the average Cq values were used for quantification. The amplification curves and dissociation curves were shown in File S1 and S2. No-template controls were used as negative control for each pair of primers. The PCR products were checked by 1.5% agarose gel electrophoresis to verify the specificity and expected sizes of amplicons. The amplification curve was analyzed by LinRegPCR for efficiency correction and baseline subtraction [37]–[39].

### Statistical Analysis

Three different types of Microsoft Excel-based software – geNorm, NormFinder, and BestKeeper – were used to rank the expression stabilities of reference genes across all of the experimental sets. The candidate reference genes were ranked by geNorm based on the expression stability value M, which was calculated for all genes investigated (the lower the M value, the higher the gene's expression stability) [35]. NormFinder program is a Visual Basic application tool for Microsoft Excel used to determine the expression stabilities of reference genes that ranks all reference gene candidates based on intra- and inter-group variations and combines both results into a stability value for each candidate reference gene [11]. BestKeeper identifies the most stably expressed genes based on the coefficient of correlation to the BestKeeper Index, which is the geometric mean of the candidate reference gene Cq values. BestKeeper also calculates the standard deviation (SD) and the coefficient of variation (CV) based on the Cq values of all candidate reference genes [36]. Genes with SD >1 are considered unacceptable [10]. Thus, with BestKeeper package the best reference genes should be the most stable ones, which should exhibit the lowest CV ± SD [40]. This analysis was done using the Cq values after efficiency correction. Following qPCR data collection, Cq values corrected after efficiency correction by LinRegPCR were converted to relative quantities using the formula: 2^−ΔCq^, in which ΔCq equals each corresponding Cq value minus the minimum Cq value. The sample with the maximum expression level (the minimum Cq value) was used as a calibrator and was set to a value of 1. Relative quantities were used for geNorm and NormFinder, while BestKeeper analysis was based on untransformed Cq values.

## Results

### Expression levels of candidate reference genes across all samples

**Figure 1:**
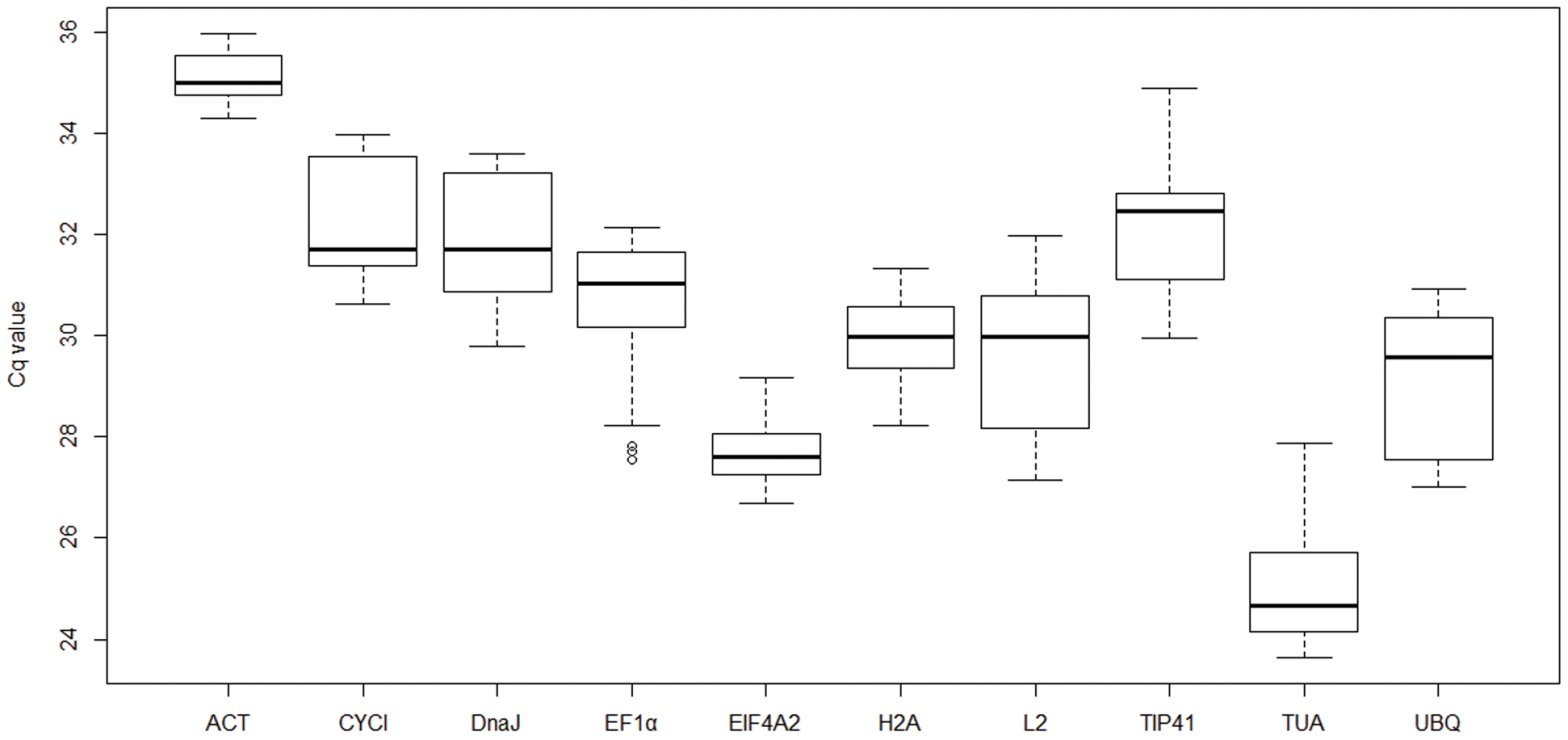
Expression levels of candidate reference genes across all samples. Lines across the boxes depict the medians, boxes indicate the interquartile range, whiskers represent 95% confidence intervals, and open circles represent outliers.

A total of 10 candidate reference genes of *R. soongorica* were chosen based on pBlast similarity against the nr database (Table 1), and their transcript levels were quantified under different stress conditions. The relative expression levels of the candidate reference genes were determined as Cq values, and the transcripts of these genes showed different levels of abundance in pooled samples (as Total), and each subset of treatments in all four genotypes (PEG, Heat, Dark, and Salt) (Figure 1). The mean Cq values of the genes ranged from 22–40, with most between 28 and 31 across all tested samples. Among all the candidate genes, *TUA* had the lowest Cq value (24.8), indicating its highest level of expression; *EIF4A2*, *H2A*, and *L2* were moderately expressed; and *ACT* was expressed at the lowest level (35.1 of Cq value). Stability was regarded as the most important characteristic for reference genes, and *ACT*, *EIF4A2 and H2A* showed the least gene expression variation (CV of 1.42%, 2.32% and 3.01% respectively), while *EF1α* (4.82%) and *TUA* (5.20%) were the most variable within the total group.

### Expression Stability of Candidate Reference Genes under different treatments geNorm analysis

**Figure 2:**
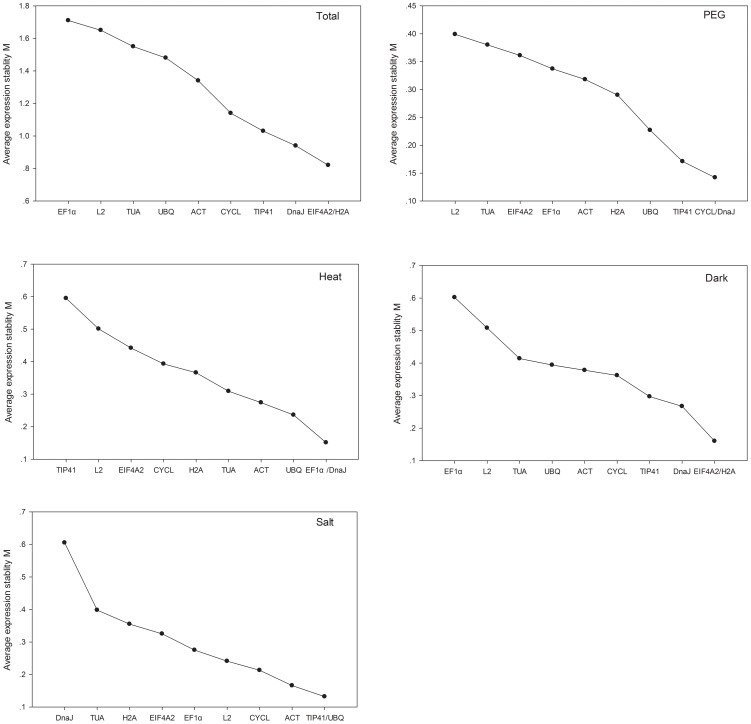
Gene expression stability and ranking of 10 reference genes calculated by geNorm. Mean expression stability (M) was calculated following stepwise exclusion of the least stable gene across all the treatment groups. The least stable genes are on the left, and the most stable ones are on the right.

The expression stabilities of the 10 reference genes were assessed using the geNorm software [35] in the pooled and separate treatments (PEG, heat, dark, and salt) across four accessions from two contrasting habitats. Figure 2 shows the ranking of the tested genes on the basis of their expression stability (M). From the pooled data, the order from the most stable gene to the least was: *H2A/EIF4A2*, *DNAJ*, *TIP41*, *CYCL*, *ACT*, *UBQ*, *TUA*, *L2*, and *EF1α* (Figure 2A). Their calculated “M” values were 0.82, 0.94, 1.03, 1.14, 1.34, 1.48, 1.55, 1.65, and 1.71, respectively. Successive elimination of the least stable genes led to the identification of *H2A* and *EIF4A2* as the most stable genes. However, we found that *CYCL* and *DNAJ* were the most stable genes in PEG treatment (Figure 2B), *EF1α* and *DNAJ* in heat (Figure 2C), *H2A* and *EIF4A2* in dark (Figure 2D), and *TIP41* and *UBQ* in salt treatment (Figure 2E). *EIF4A2* was the moderate stable gene in individual PEG, heat, and salt treatments, which is inconsistent with the CV analysis of the pooled data.

**Figure 3:**
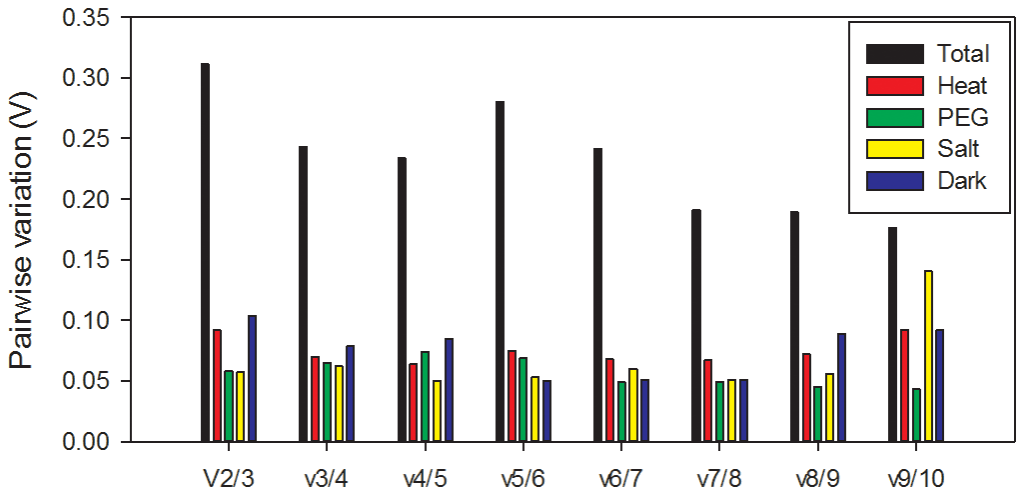
Determination of the optimal number of reference genes required for effective normalization. Pairwise variation (Vn/Vn+1) analysis between the normalization factors (NFn and NFn+1) was performed by the geNorm program to determine the optimal number of reference genes and was carried out for qPCR data normalization in various sample pools.

geNorm also performs stepwise calculations of the pair-wise variation (Vn/Vn+1) between sequential normalization factors (NFn and NFn+1) to determine the optimal number of reference genes required for accurate normalization. A large variation means that the added gene had a significant effect and should preferably be included to calculate a reliable normalization factor [35]. The cut-off value of 0.15 was proposed by the geNorm program [35]; below this cut-off value there is no need of inclusion of an additional control gene. As shown in Figure 3, when all the samples were taken into account, the pairwise variation V2/3 was 0.311, higher than 0.15, indicating that the addition of the third reference gene was necessary to normalize gene expression. *H2A* and *EIF4A2* were recommended as the best reference genes based on their M value (Figure 2A). However, for individual treatments, V2/3 values were 0.092, 0.058, 0.057, and 0.104 in Heat, PEG, Salt, and Dark treatments, respectively. It is apparent that the inclusion of a third gene did not have a significant effect in separate treatments.

**Figure 4:**
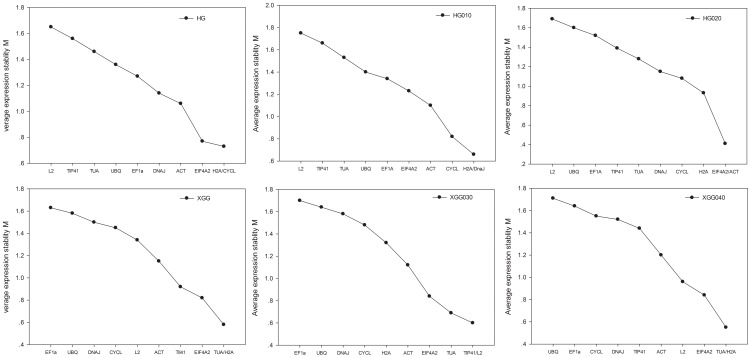
Expression stability and ranking of housekeeping genes by geNorm analysis within and among populations. Mean expression stability (M) was calculated following stepwise exclusion of the least stable gene.

The expression stabilities of the 10 reference genes were also assessed among genotypes (Figure 4). In the HG genotypes (HG010 and HG020), the *CYCL*, *H2A* and *EIF4A2* were most stable; while in XGG genotypes (XGG030 and XGG040) were *EIF4A2*, *H2A* and *TUA*. Among individual genotypes, *H2A* and *EIF4A2* were frequently observed in the top of the most sable genes. However, compared to *H2A* and *EIF4A2*, *ACT* and *UBQ* which were usually used as reference gene for qPCR were not a good choice.

### NormFinder analysis

NormFinder analysis results (shown in Table 2) revealed that the expression of three candidate reference genes, *EIF4A2*, *ACT*, and *H2A* had lower stability values (the lower, the better) across all the samples pooled together. However, the stability ranking of genes in individual treatments were significantly different; for example, the best reference genes were found as *UBQ* in PEG, *EF1α* and *DNAJ* in Heat, *DNAJ* in Dark, and *UBQ* and *CYCL* in Salt, and all the genes harbored the stable values <0.1. Interestingly, according to NormFinder analysis, *ACT* was suggested as a good reference gene among the four treatments, which is inconsistent with the conclusion from the geNorm analysis. However, the results from the NormFinder and geNorm analyses were consistent, suggesting that *EIF4A2* and *H2A* are stable reference genes.

**Table 2:** Expression stability of the reference genes calculated by NormFinder software.

Total	SV	PEG	SV	Heat	SV	Dark	SV	Salt	SV
*EIF4A2*	0.368	*UBQ*	0.073	*EF1α*	0.053	*DNAJ*	0.093	*UBQ*	0.043
*ACT*	0.4	*CYCL*	0.144	*DNAJ*	0.062	*TIP41*	0.147	*CYCL*	0.063
*H2A*	0.573	*DNAJ*	0.186	*ACT*	0.203	*ACT*	0.173	*TIP41*	0.11
*DNAJ*	0.677	*H2A*	0.19	*TUA*	0.216	*EIF4A2*	0.198	*TUA*	0.203
*CYCL*	0.874	*TIP41*	0.212	*UBQ*	0.238	*CYCL*	0.215	*L2*	0.209
*TUA*	1.017	*EF1α*	0.212	*CYCL*	0.244	*UBQ*	0.254	*EF1α*	0.21
*UBQ*	1.046	*ACT*	0.231	*H2A*	0.253	*TUA*	0.302	*ACT*	0.235
*TIP41*	1.119	*EIF4A2*	0.234	*EIF4A2*	0.382	*H2A*	0.311	*H2A*	0.32
*L2*	1.157	*TUA*	0.287	*L2*	0.451	*L2*	0.515	*EIF4A2*	0.329
*EF1α*	1.185	*L2*	0.291	*TIP41*	0.632	*EF1α*	0.628	*DNAJ*	0.978

Note: SV, stability value.

### BestKeeper analysis

The results of the BestKeeper analysis are shown in Table 3. Four candidate reference genes (*EIF4A2*, *ACT*, *H2A*, and *DNAJ*) in the Total group showed an SD value <1, which qualified as reference genes. When taking into account the separate treatments, all the candidate genes showed SD values <1 in individual groups. Here, only the top two genes were listed, as *UBQ* and *CYCL* in PEG treatment, *EF1α* and *DNAJ* in heat treatment, *DNAJ* and *TIP41* in dark treatment, and *UBQ*, *CYCL* in salt treatment. Further data processing using Pearson correlation coefficient and regression analysis (r) showed that *ACT* exhibited the lowest correlation with other candidate genes (data not shown, r = −0.676, p-value = 0.001), suggesting its higher independence. *DNAJ* was the most correlated to the other nine genes in pool (r = 0.68, p-value = 0.001). Pairwise correlation revealed that *TIP41* and *TUA* (r = 0.804, p-value = 0.001) were highly correlated. As these two genes were not stable in pooled data, we did not take these two genes as good reference. Although *EIF4A2* and *H2A* was the second most correlated gene pair (r = 0.773, p-value = 0.001), considering their higher scores in geNorm and NormFinder, *EIF4A2* and *H2A* could be selected as two likely reference genes with good correlation.

**Table 3:** Expression stability of the reference genes calculated by BestKeeper software.

Total	SD	CV	PEG	SD	CV	Heat	SD	CV	Dark	SD	CV	Salt	SD	CV
*ACT*	0.43	1.22	*ACT*	0.09	0.26	*EF1α*	0.12	0.39	*ACT*	0.19	0.54	*L2*	0.08	0.26
*EIF4A2*	0.49	1.75	*H2A*	0.12	0.4	*DNAJ*	0.12	0.39	*DNAJ*	0.2	0.63	*UBQ*	0.12	0.4
*H2A*	0.74	2.47	*UBQ*	0.19	0.69	*ACT*	0.18	0.51	*TIP41*	0.23	0.76	*EF1α*	0.14	0.45
*DNAJ*	0.98	3.07	*EF1α*	0.2	0.73	*H2A*	0.21	0.71	*TUA*	0.23	0.94	*TIP41*	0.16	0.48
*TUA*	1	4.01	*L2*	0.26	0.84	*CYCL*	0.22	0.71	*EIF4A2*	0.24	0.87	*CYCL*	0.16	0.52
*TIP41*	1.07	3.32	*TIP41*	0.28	0.8	*UBQ*	0.24	0.78	*CYCL*	0.25	0.73	*ACT*	0.22	0.62
*CYCL*	1.08	3.33	*CYCL*	0.33	1.05	*TUA*	0.25	1.01	*H2A*	0.34	1.11	*EIF4A2*	0.3	1.08
*EF1α*	1.16	3.78	*DNAJ*	0.37	1.22	*EIF4A2*	0.38	1.39	*UBQ*	0.35	1.27	*H2A*	0.39	1.28
*UBQ*	1.26	4.33	*EIF4A2*	0.51	1.79	*L2*	0.45	1.62	*EF1α*	0.56	1.79	*TUA*	0.4	1.61
*L2*	1.33	4.5	*TUA*	0.58	2.13	*TIP41*	0.74	2.32	*L2*	0.69	2.44	*DNAJ*	1.24	3.86

Note: Reference genes were identified as the most stable genes, i.e. those with the lowest coefficient of variance (CV) with standard deviation (SD).

### Reference Gene Validation

**Figure 5:**
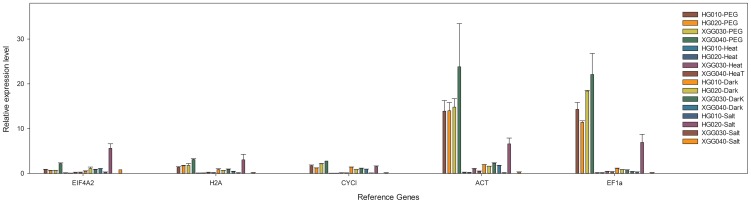
Relative quantification of *rbcL* expression using 10 reference genes for normalization in the four accessions. The results are represented as mean fold changes in relative expression compared to untreated samples.

To validate candidate reference gene selection, the expression pattern of *rbcL* was analyzed against all 10 candidate reference genes (Figure 5). RuBisCO is an enzyme involved in the first major step of carbon fixation, a process by which atmospheric carbon dioxide is converted by plants to energy-rich molecules, such as glucose. In chemical terms, it catalyzes the carboxylation of ribulose-1,5-bisphosphate (also known as RuBP). It is probably the most abundant protein on Earth [41], [42]. It was obviously down-regulated due to its light-dependent characteristic, and *rbcL* has also been shown to be down-regulated in soybean under stress [43], [44]. In our results, *rbcL* was down-regulated against all genes except PEG treatments in three accessions (HG010, XGG030, and XGG040). However, in PEG treatments, *rbcL* was slightly down-regulated against *EF1α*, *CYCL*, *H2A*, *DNAJ*, and *UBQ*, which were the most stable genes in PEG. It was strongly up-regulated compared to *TUA*, *ACT*, *EIF4A2*, and *L2*, which were the most unstable genes in PEG treatment. In accession HG020, It was obvious that its expression level was down-regulated against most candidate reference genes, except in PEG and salt treatments. This could be due to the divergent genetic backgrounds between this genotype and the other three genotypes. Actually, the huge genetic differentiation within populations was supported by a phylogeography study based on chloroplast genes (unpublished data), and different genotypes may exhibit different physiological responses under drought conditions. Thus, the high expression level of *rbcL* in HG020 accession may be related to local environment adaptation. Except this, the validation results against the most stable genes were all consistent with the results from soybean [43], [44], suggesting that the *DNAJ* and *H2A* genes could be used as practical reference genes in multiple treatments.

## Discussion

qPCR has significantly improved the detection and quantification of expression profiles of genes in distinct biological samples. The main advantages of this technique are based on its high sensitivity, high specificity, and broad quantification range [45] [46]. For these reasons, qPCR is the first choice for detecting and quantifying gene expression profiles. An appropriate internal control gene is required for valid qPCR analyses. The ideal control gene should have relatively stable expression in distinct biological samples, which can be from different cell types, developmental stages, and samples exposed to different experimental conditions, even for different genotypes.

*R. soongorica* is regarded as a plant that is tolerant to extremely drought conditions [26]–[28]. Moreover, *R. soongorica* is a typical recretohalophyte, and its molecular mechanism of salt resistance remains unclear [47]. Only a few studies have focused on the molecular mechanisms of adaption to extreme environments [27]. Since his shrub species is prevalent in ecological engineering and restoration to mitigate further desertification of the arid land in northwest China, with its numerous novel genetic resources (around 40% of genes were newly found in its transcriptome sequences [34]),, it is convenient and feasible to focus on molecular functional analyses of the expression of unique genes. However, it is first necessary to identify stable reference genes.

In this study, the four stresses of drought, salt, dark, and heat were investigated in different *R. soongorica* genotypes to identify stable reference genes. Three different statistical approaches (geNorm, NormFinder, and BestKeeper) were used to evaluate the stability of the 10 most commonly used candidate reference genes. The most prominent observation was that each type of software produced a different set of top-ranked reference genes. This is not unexpected because the three programs employed different algorithms and analytical procedures. The top three stable reference genes were *EIF4A2*, *H2A*, and *DNAJ* using geNorm. *EIF4A2*, *ACT*, and *H2A* had lower stability values across all samples using NormFinder, which was consistent with the BestKeeper results. We used a previously described method [48] to determine the best reference gene based on all three algorithms (Table S2). According to the frequencies of the reference genes in the rank crossing the difference treatments (PEG, Salt, Dark, Heat and Total), *H2A* and *EIF4A2* were the best reference genes, *CYCL* was moderate stable, and *EF1α* was the fourth choice. This was supported in the subsequent validation analysis with *rbcL* (File S4). All the 10 genes are normally expressed under all the treatments in the 4 genotypes (Figure S2), indicating that normalization of total RNA was sufficient. In PEG treatments, *rbcL* was slightly or down-regulated against *EF1α*, *CYCL*, *H2A*, *DNAJ*, and *UBQ*, which were the most stable genes in accordance with results in Soybean [43], [44]. Once we identify practical internal reference gene, it is easy to characterize the true expression level for a candidate gene.

*ACT* has been considered as one of the best reference genes for assessing gene expression in many plant tissues and under different experimental conditions [49]–[51]. In present study, GeNorm, NormFinder, and Bestkeeper analyses demonstrated that *ACT* was not that stable and exhibited a lower expression level compared to other good reference genes, which suggested that it is not a good reference gene for *R. soongorica* under such conditions. Actually, the instablity of *ACT* homolog genes was also confirmed in two other plant species [12], [15]. The *ACT* homolog gene exhibited similar low expression levels in *Caragana intermedia* and *R. soongorica*, which are widely spread throughout the arid zone in China. Even though *C. intermedia* and *R. soongorica* belong to different families, it is likely that both of them harbor similar pathways involved in strong drought- and salt-resistance ability.

The other two most commonly used reference genes are *UBQ* and *TUA*. *UBQ* showed highly stable expression in Arabidopsis [52] and tomato [9], but the *UBQ* gene was ranked differently across different treatments in the present study. It was listed on the top under PEG with NormFinder; however, it was in a moderate stable position with geNorm and BestKeeper under other treatments. It was suggested to be an inappropriate internal control for qPCR studies at different developmental stages in rice [53] and soybean [54]. *TUA* has also been widely used as a reference gene in studies in water lily and soybean [16], [19]. However, some studies revealed that *TUA* did not satisfy certain basic requirements for use as an internal control [18]. In our results in *R. soongorica*, *TUA* was once of the least stable genes according to geNorm, BestKeeper, and NormFinder analyses together with *rbcL* expression level. Consequently, *ACT*, *UBQ*, and *TUA* should be used with caution as an internal control in *R. soongorica*.

## Conclusions

To our knowledge, this study is the first to systematically analyze reference genes for qPCR in *R. soongorica* under different abiotic stress conditions (drought, salt, dark, and heat) among four accessions. Analysis of expression stability using geNorm, NormFinder, and BestKeeper revealed that *EIF4A2* and *H2A* were appropriate reference genes under different abiotic stress conditions, whereas *EF1α* showed relatively low expression stability. In contrast, the most commonly used reference genes *ACT*, *UBQ*, and *TUA* were inappropriate. This work will benefit future gene expression studies assessing different abiotic stress conditions in *R. soongorica* and other *Reaumuria* species.

## Supporting Information

Figure S1 **Agarose gel electrophoresis for PCR products of 10 candidate reference genes and** ***rbcL*** **.**
(TIFF)

Figure S2 **Expression levels of candidate reference genes across all the four accessions.**
(TIFF)

Table S1 **Annotation information of 10 reference genes and the one validation gene from the** ***de novo*** **transcriptome sequences.**
(XLS)

Table S2 **Ranking of the best reference gene based on the all three algorithms.**
(DOC)

File S1 **Contains 11 figures from Figure S3 to S13 representing the qPCR amplification curves of 10 candidate reference genes (** ***ACT, CYCL, DNAJ, EF1, EIF4A2, H2A, L2, TIP41, TUA and UBQ*** **) and one validation gene** ***rbcL*** **, respectively.**
(ZIP)

File S2 **Contains 11 figures from Figure S14 to S24 representing the melting curves of qPCR products of the 10 candidate reference genes (** ***ACT, CYCL, DNAJ, EF1, EIF4A2, H2A, L2, TIP41, TUA and UBQ*** **) and one validation gene** ***rbcL*** **, respectively.**
(ZIP)

File S3 **Alignment of the protein sequences of** ***rbcL*** **homologs.**
(PDF)

File S4 **Contains 11 sequence alignment files from Fasta S1 to S11,for verification of the homologs of 10 reference genes and one validation gene** ***rbcL*** **.**
(ZIP)
