# Supplementary figures and images for: Reference Gene Selection for Quantitative Real-Time PCR Normalization in Reaumuria soongorica
Source: PLoS One. 2014 Aug 12;9(8):e104124. doi: 10.1371/journal.pone.0104124 (PMC4130609; doi:10.1371/journal.pone.0104124)

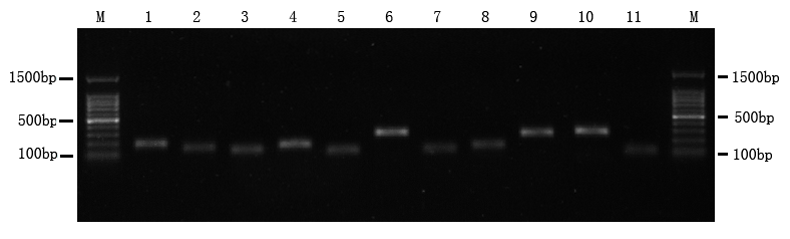

Supplement: Figure S1 — Agarose gel electrophoresis for PCR products of 10 candidate reference genes and rbcL . (TIFF) [file pone.0104124.s001.tiff]

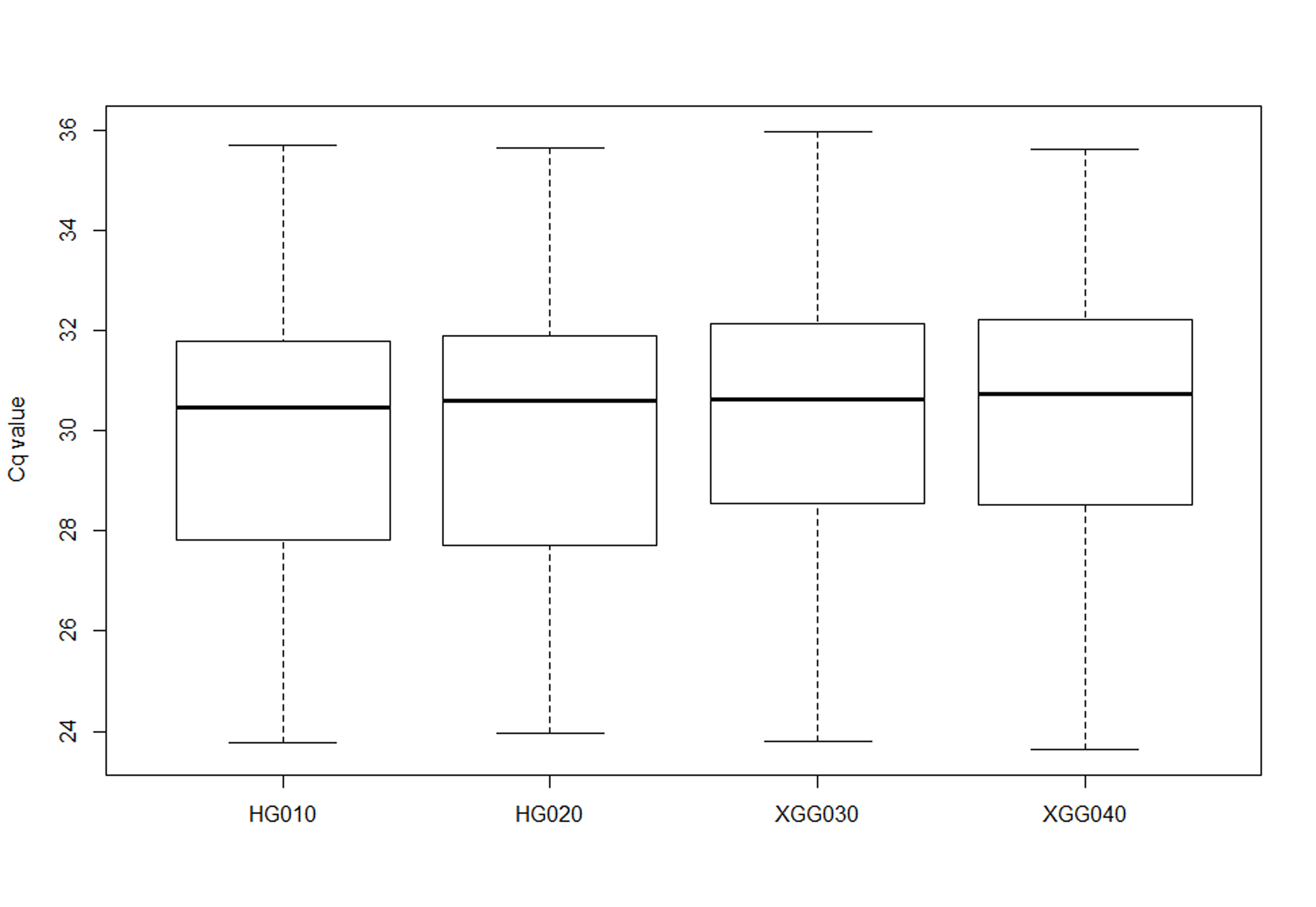

Supplement: Figure S2 — Expression levels of candidate reference genes across all the four accessions. (TIFF) [file pone.0104124.s002.tiff]

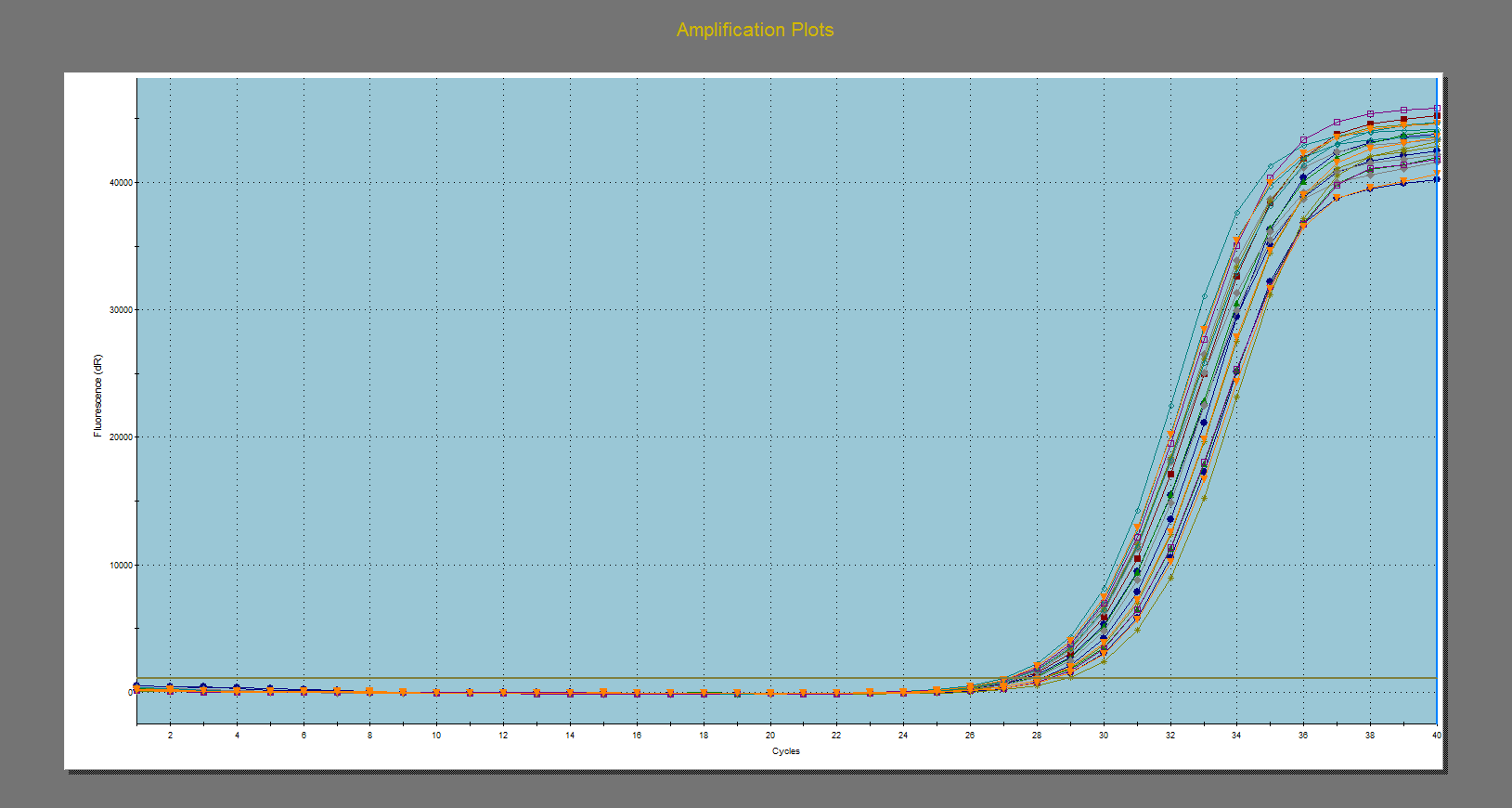

Supplement: File S1 — Contains 11 figures from Figure S3 to S13 representing the qPCR amplification curves of 10 candidate reference genes ( ACT, CYCL, DNAJ, EF1, EIF4A2, H2A, L2, TIP41, TUA and UBQ ) and one validation gene rbcL , respectively. (ZIP) [file pone.0104124.s005.zip › File S1/Figure S10 TIP41.bmp]

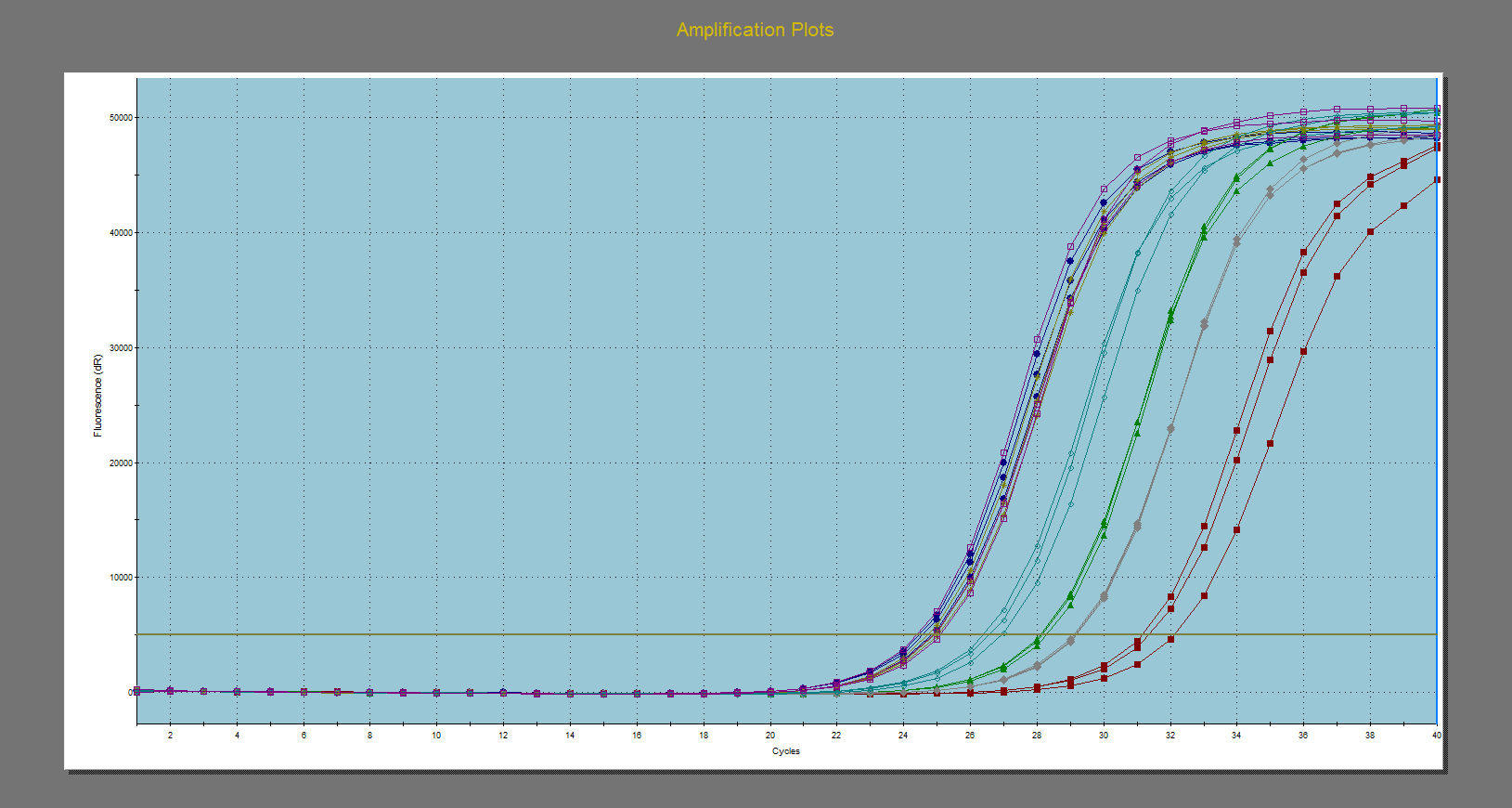

Supplement: File S1 — Contains 11 figures from Figure S3 to S13 representing the qPCR amplification curves of 10 candidate reference genes ( ACT, CYCL, DNAJ, EF1, EIF4A2, H2A, L2, TIP41, TUA and UBQ ) and one validation gene rbcL , respectively. (ZIP) [file pone.0104124.s005.zip › File S1/Figure S11 TUA.bmp]

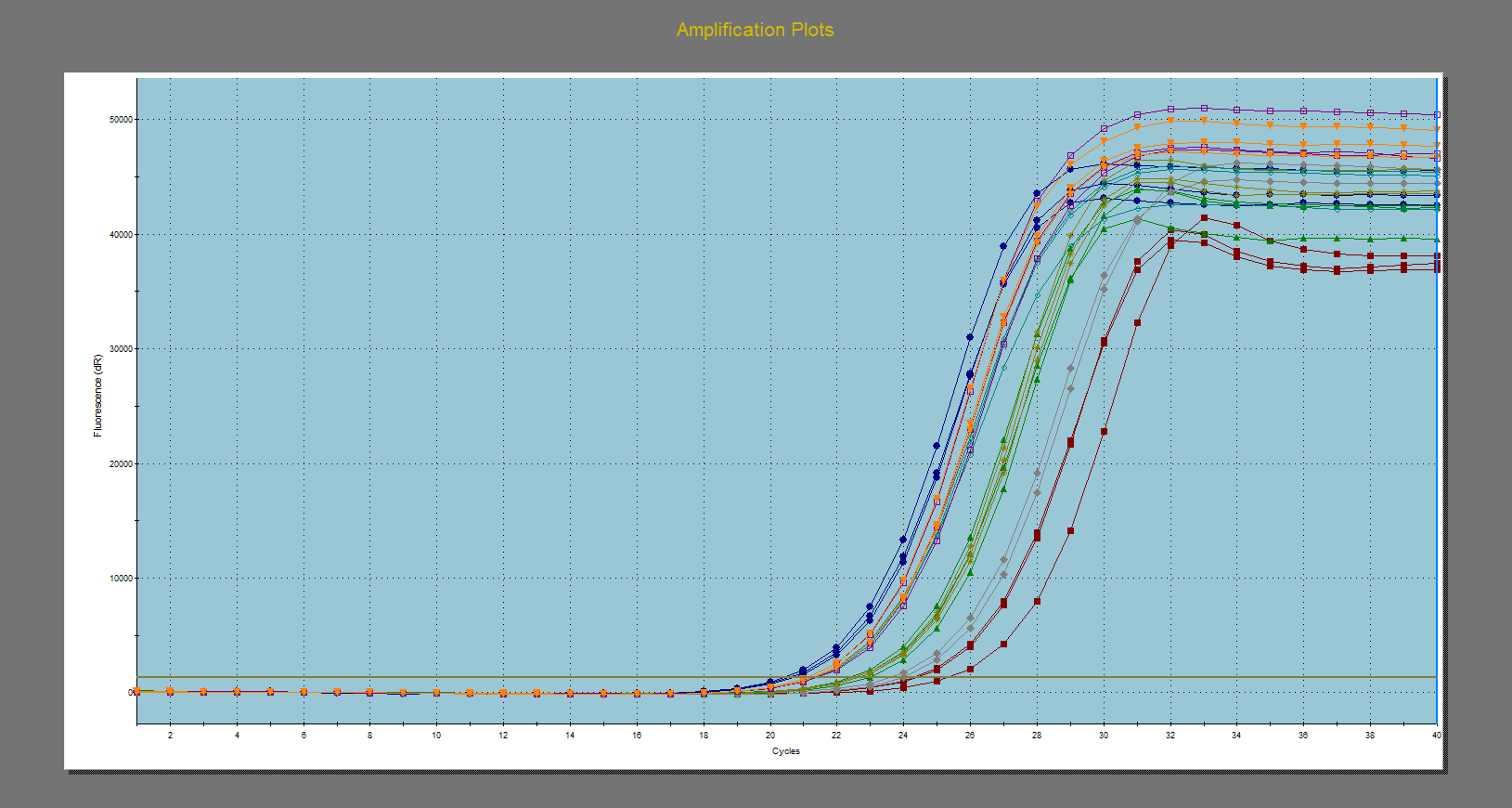

Supplement: File S1 — Contains 11 figures from Figure S3 to S13 representing the qPCR amplification curves of 10 candidate reference genes ( ACT, CYCL, DNAJ, EF1, EIF4A2, H2A, L2, TIP41, TUA and UBQ ) and one validation gene rbcL , respectively. (ZIP) [file pone.0104124.s005.zip › File S1/Figure S13 rbcL.bmp]

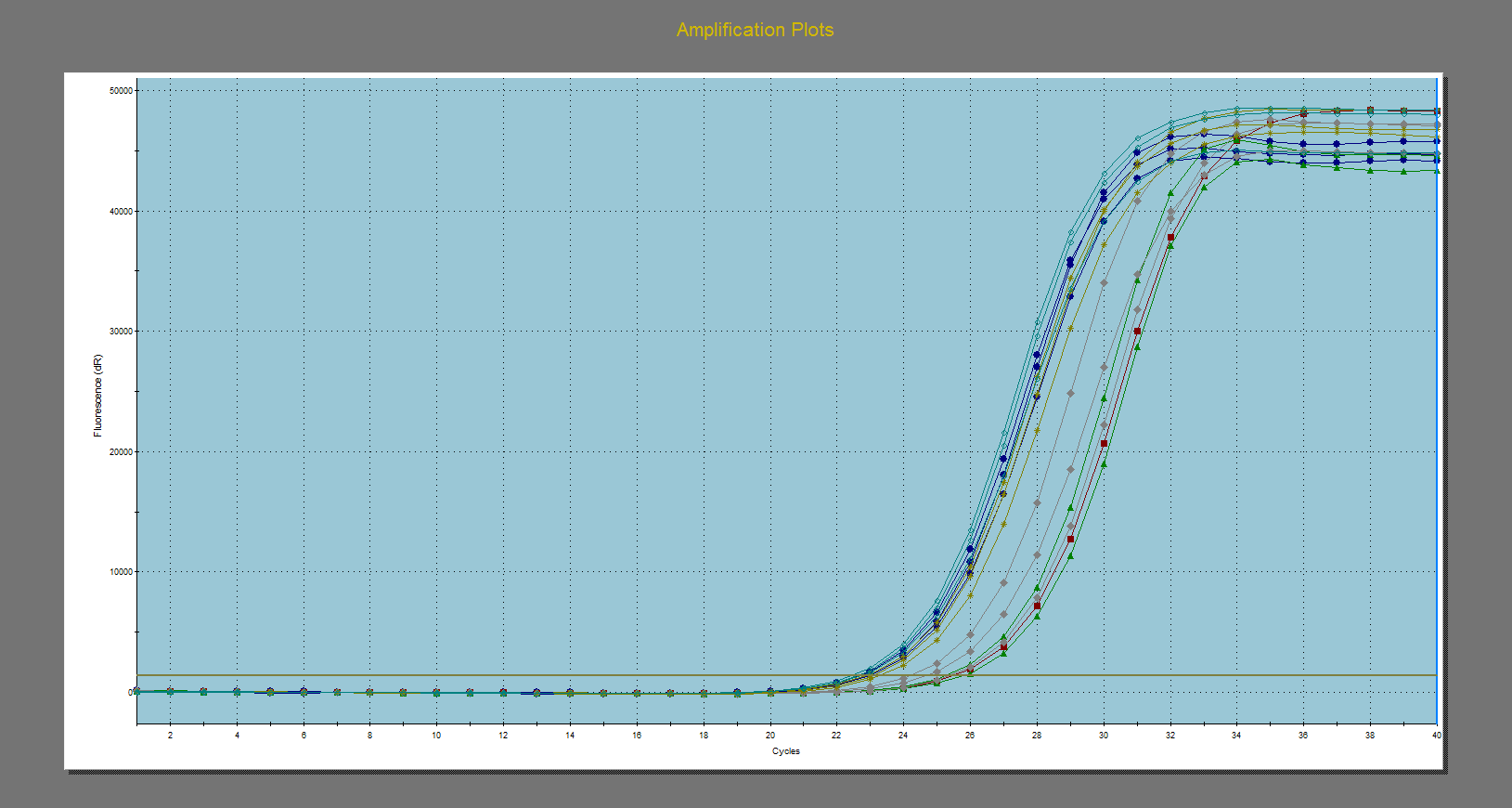

Supplement: File S1 — Contains 11 figures from Figure S3 to S13 representing the qPCR amplification curves of 10 candidate reference genes ( ACT, CYCL, DNAJ, EF1, EIF4A2, H2A, L2, TIP41, TUA and UBQ ) and one validation gene rbcL , respectively. (ZIP) [file pone.0104124.s005.zip › File S1/Figure S3 ACT.bmp]

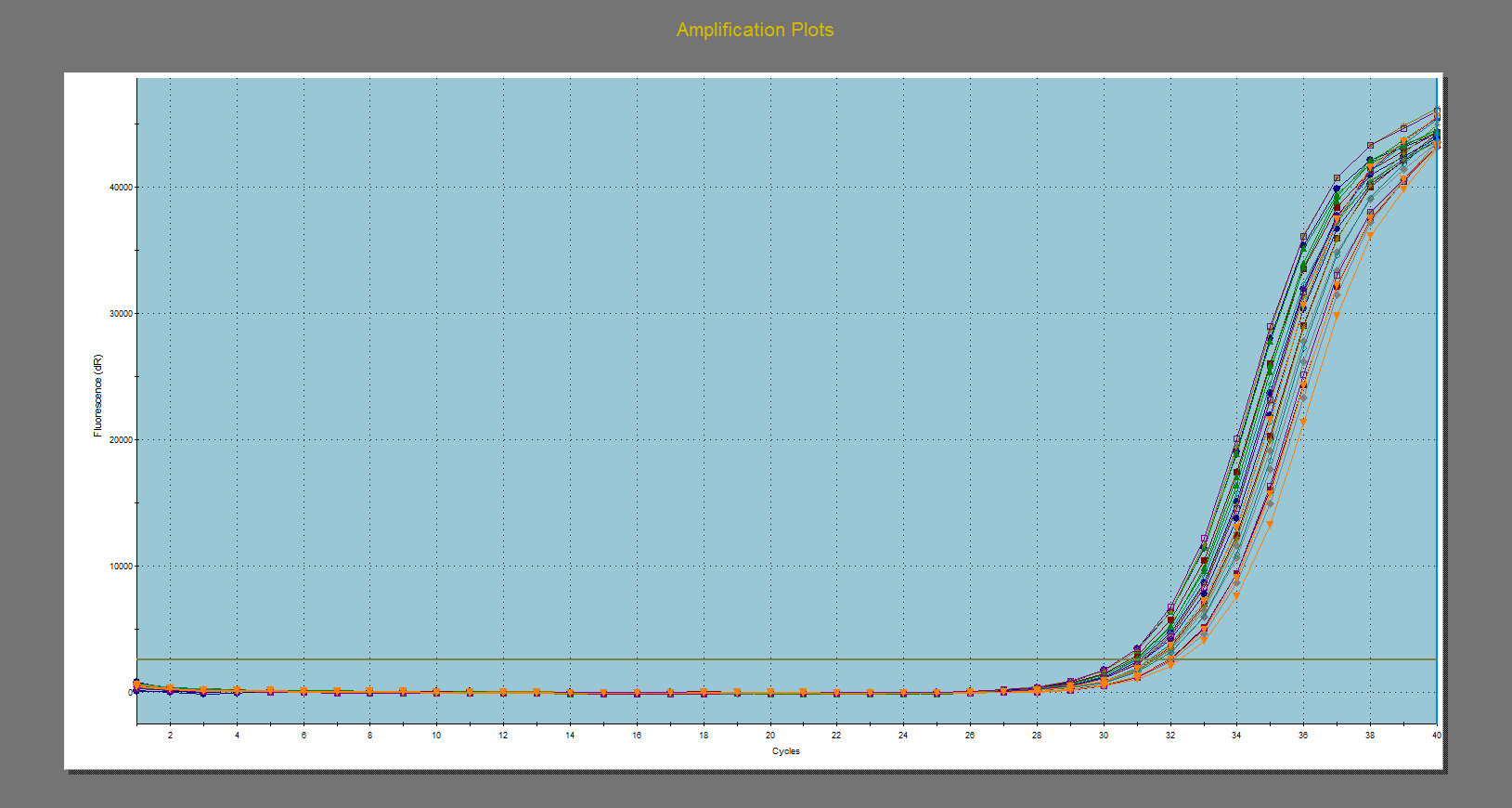

Supplement: File S1 — Contains 11 figures from Figure S3 to S13 representing the qPCR amplification curves of 10 candidate reference genes ( ACT, CYCL, DNAJ, EF1, EIF4A2, H2A, L2, TIP41, TUA and UBQ ) and one validation gene rbcL , respectively. (ZIP) [file pone.0104124.s005.zip › File S1/Figure S4 CYCL.bmp]

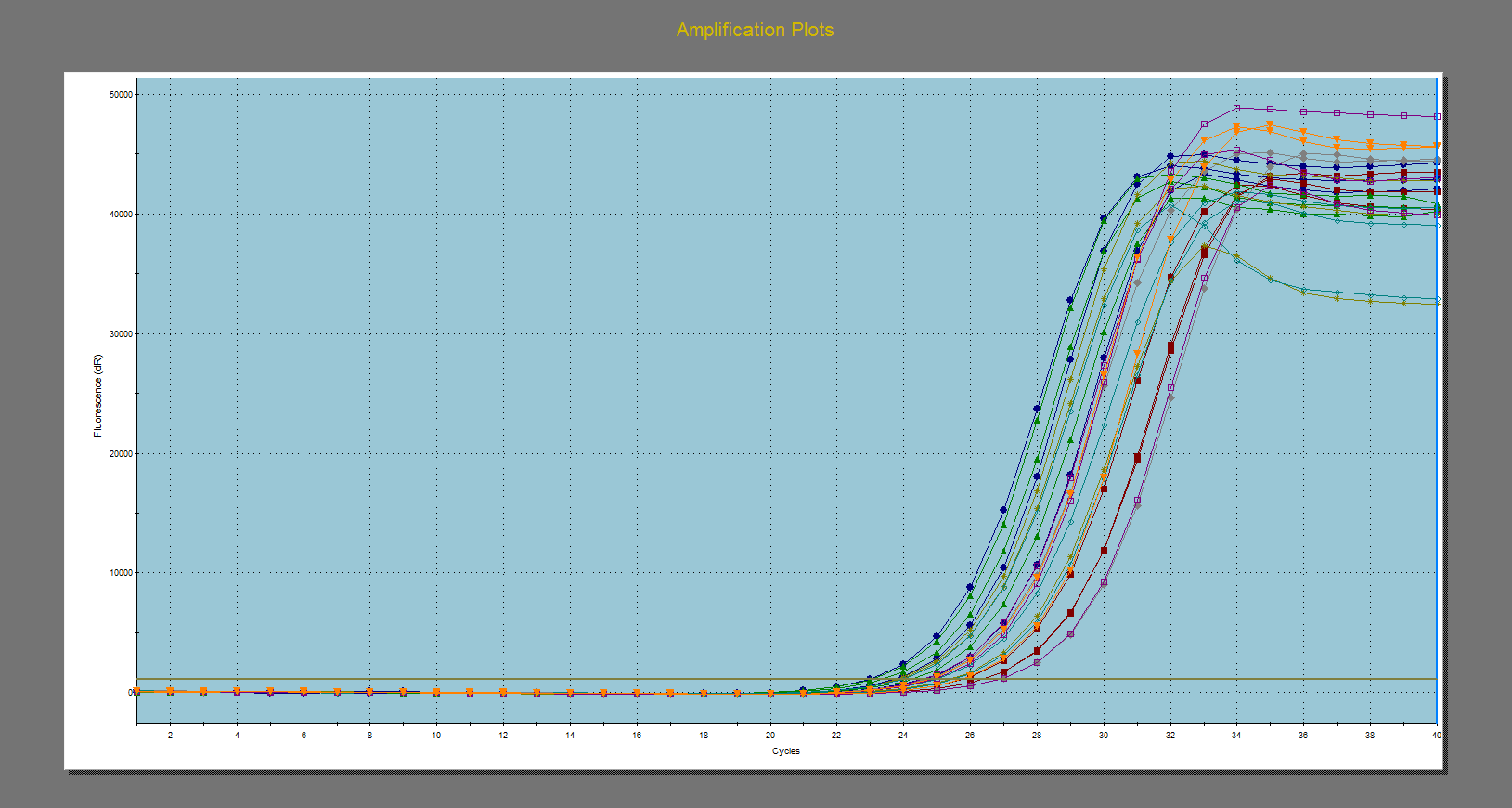

Supplement: File S1 — Contains 11 figures from Figure S3 to S13 representing the qPCR amplification curves of 10 candidate reference genes ( ACT, CYCL, DNAJ, EF1, EIF4A2, H2A, L2, TIP41, TUA and UBQ ) and one validation gene rbcL , respectively. (ZIP) [file pone.0104124.s005.zip › File S1/Figure S5 DNAJ.bmp]

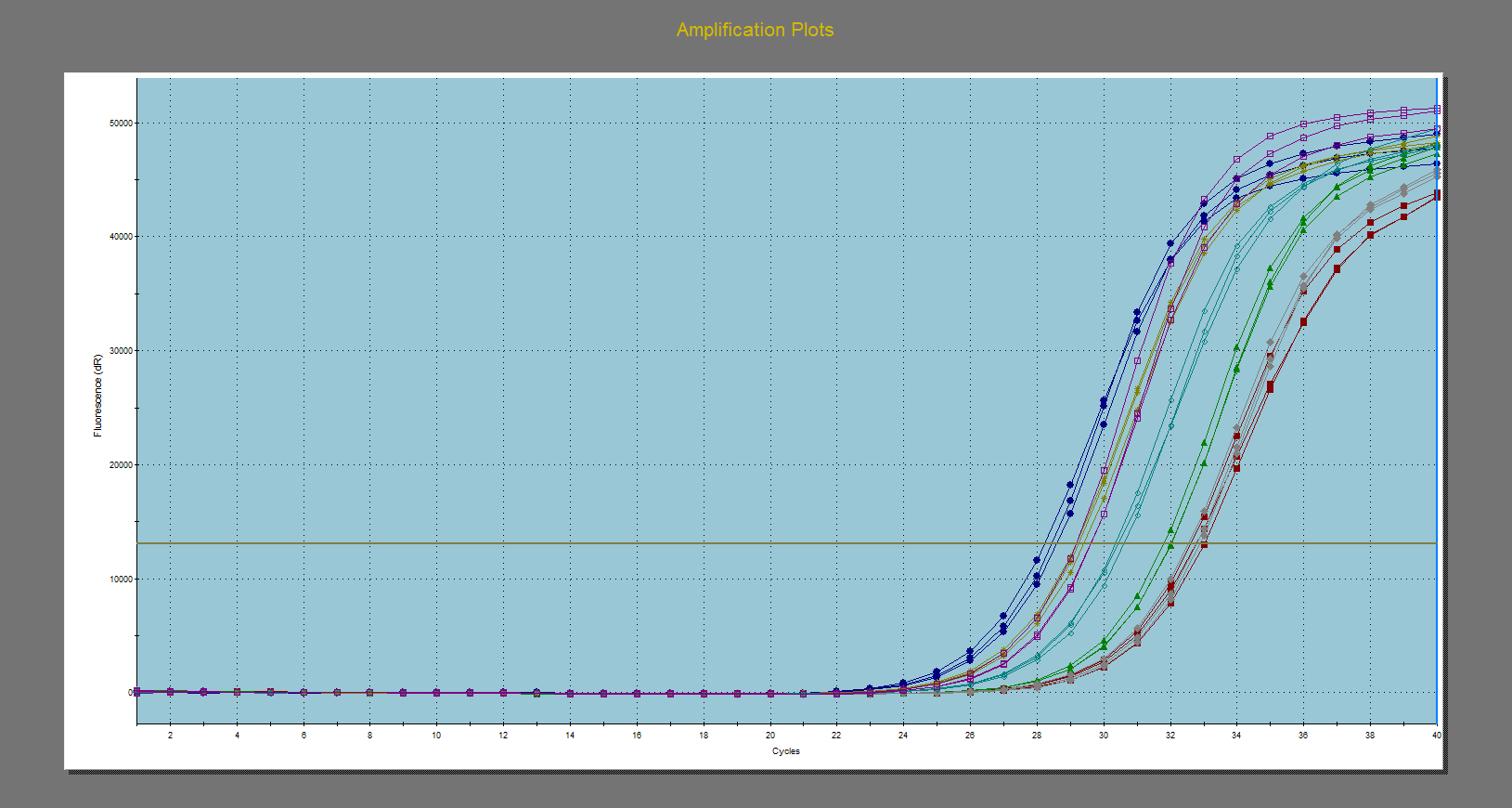

Supplement: File S1 — Contains 11 figures from Figure S3 to S13 representing the qPCR amplification curves of 10 candidate reference genes ( ACT, CYCL, DNAJ, EF1, EIF4A2, H2A, L2, TIP41, TUA and UBQ ) and one validation gene rbcL , respectively. (ZIP) [file pone.0104124.s005.zip › File S1/Figure S6 EF1a.bmp]

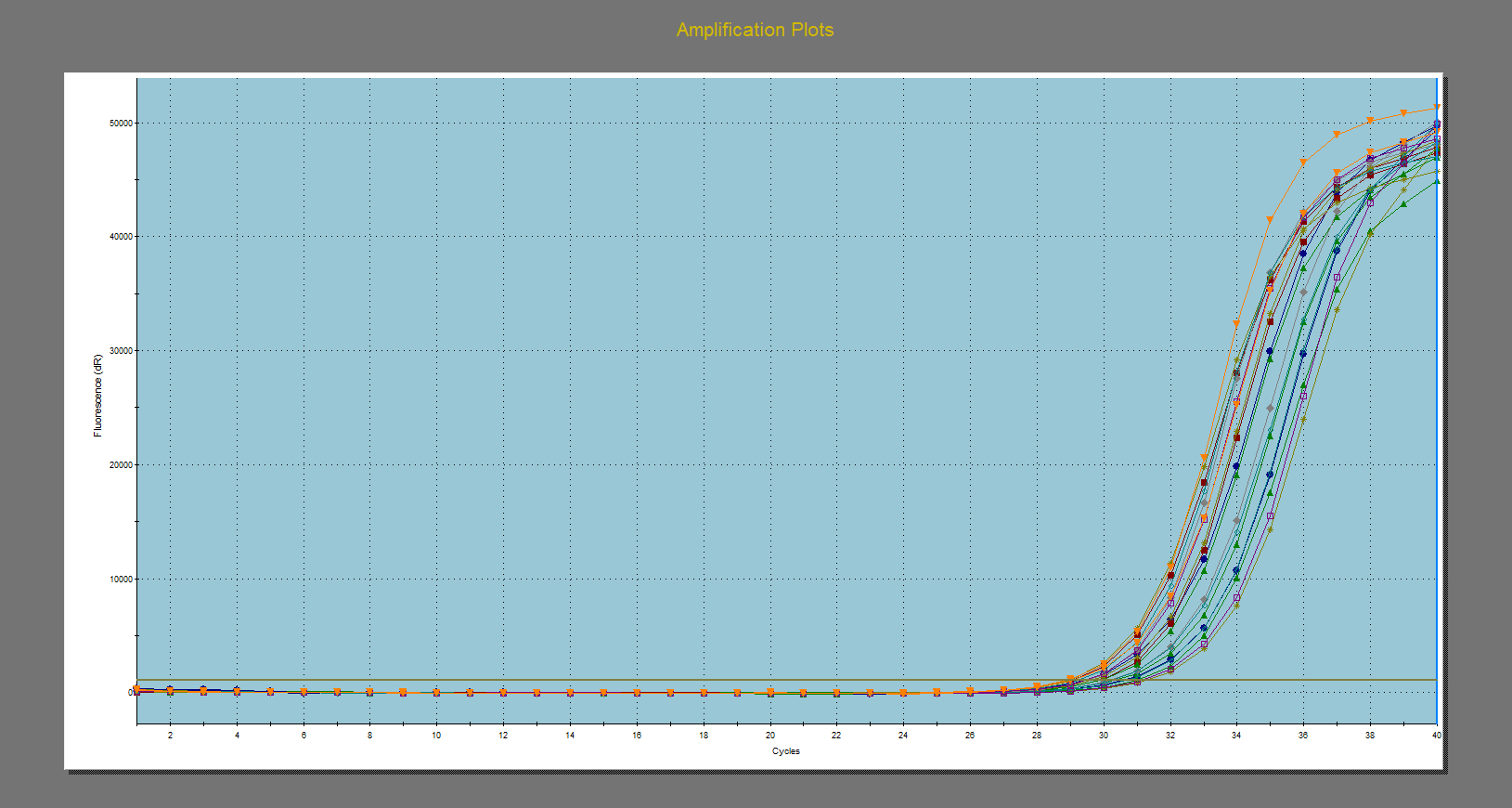

Supplement: File S1 — Contains 11 figures from Figure S3 to S13 representing the qPCR amplification curves of 10 candidate reference genes ( ACT, CYCL, DNAJ, EF1, EIF4A2, H2A, L2, TIP41, TUA and UBQ ) and one validation gene rbcL , respectively. (ZIP) [file pone.0104124.s005.zip › File S1/Figure S7 EIF4A2.bmp]

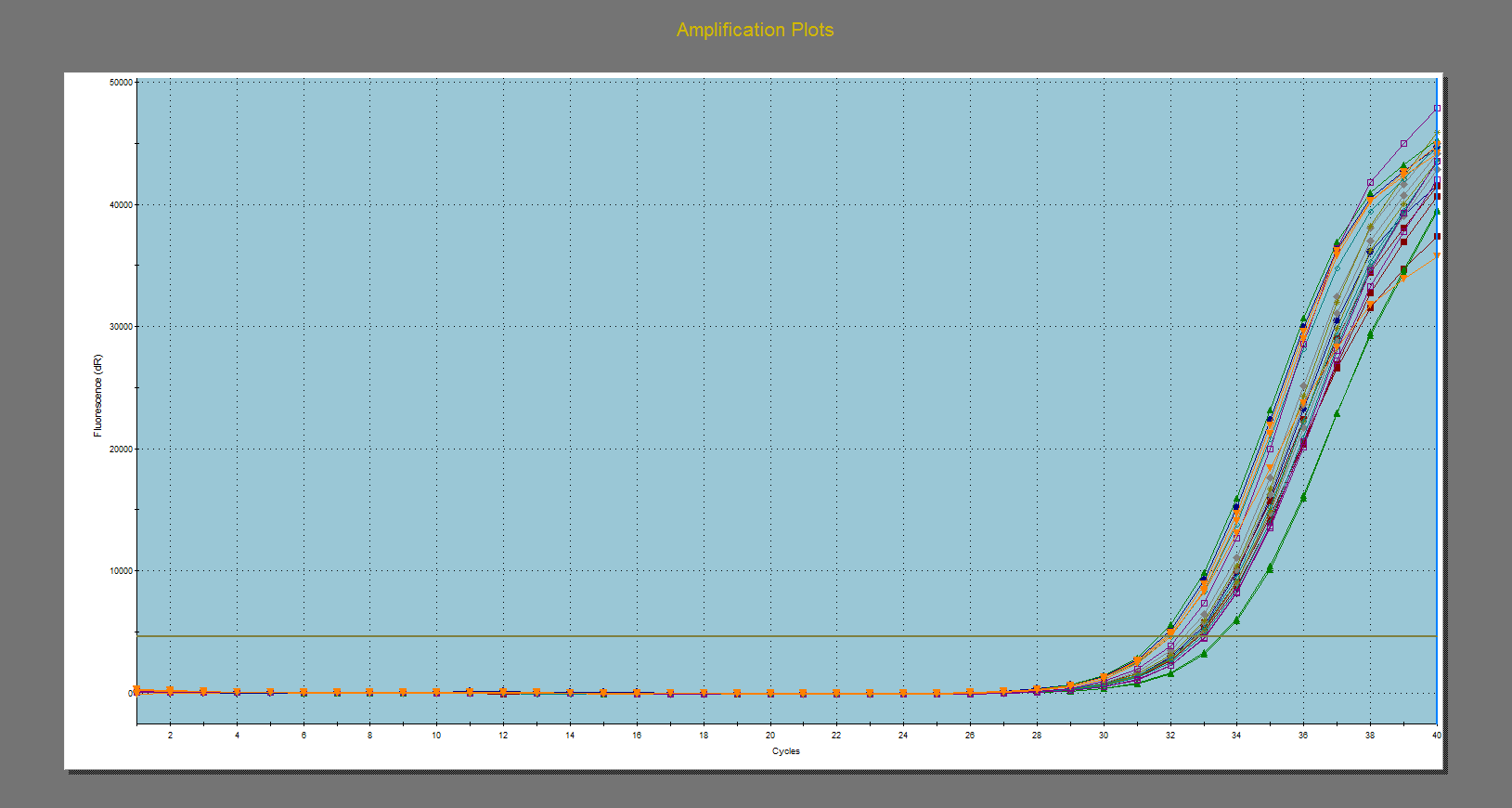

Supplement: File S1 — Contains 11 figures from Figure S3 to S13 representing the qPCR amplification curves of 10 candidate reference genes ( ACT, CYCL, DNAJ, EF1, EIF4A2, H2A, L2, TIP41, TUA and UBQ ) and one validation gene rbcL , respectively. (ZIP) [file pone.0104124.s005.zip › File S1/Figure S8 H2A.bmp]

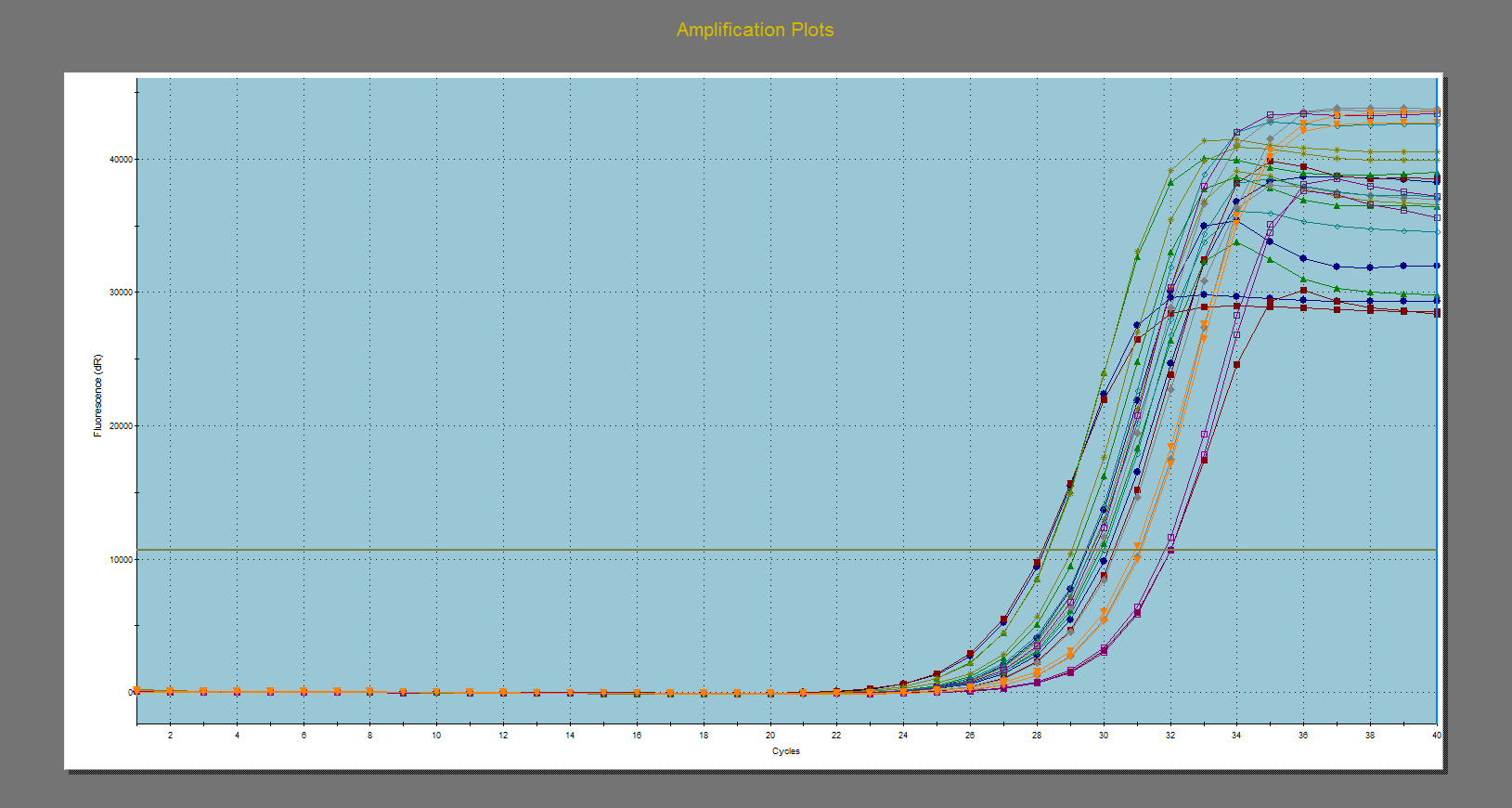

Supplement: File S1 — Contains 11 figures from Figure S3 to S13 representing the qPCR amplification curves of 10 candidate reference genes ( ACT, CYCL, DNAJ, EF1, EIF4A2, H2A, L2, TIP41, TUA and UBQ ) and one validation gene rbcL , respectively. (ZIP) [file pone.0104124.s005.zip › File S1/Figure S9 L2.bmp]

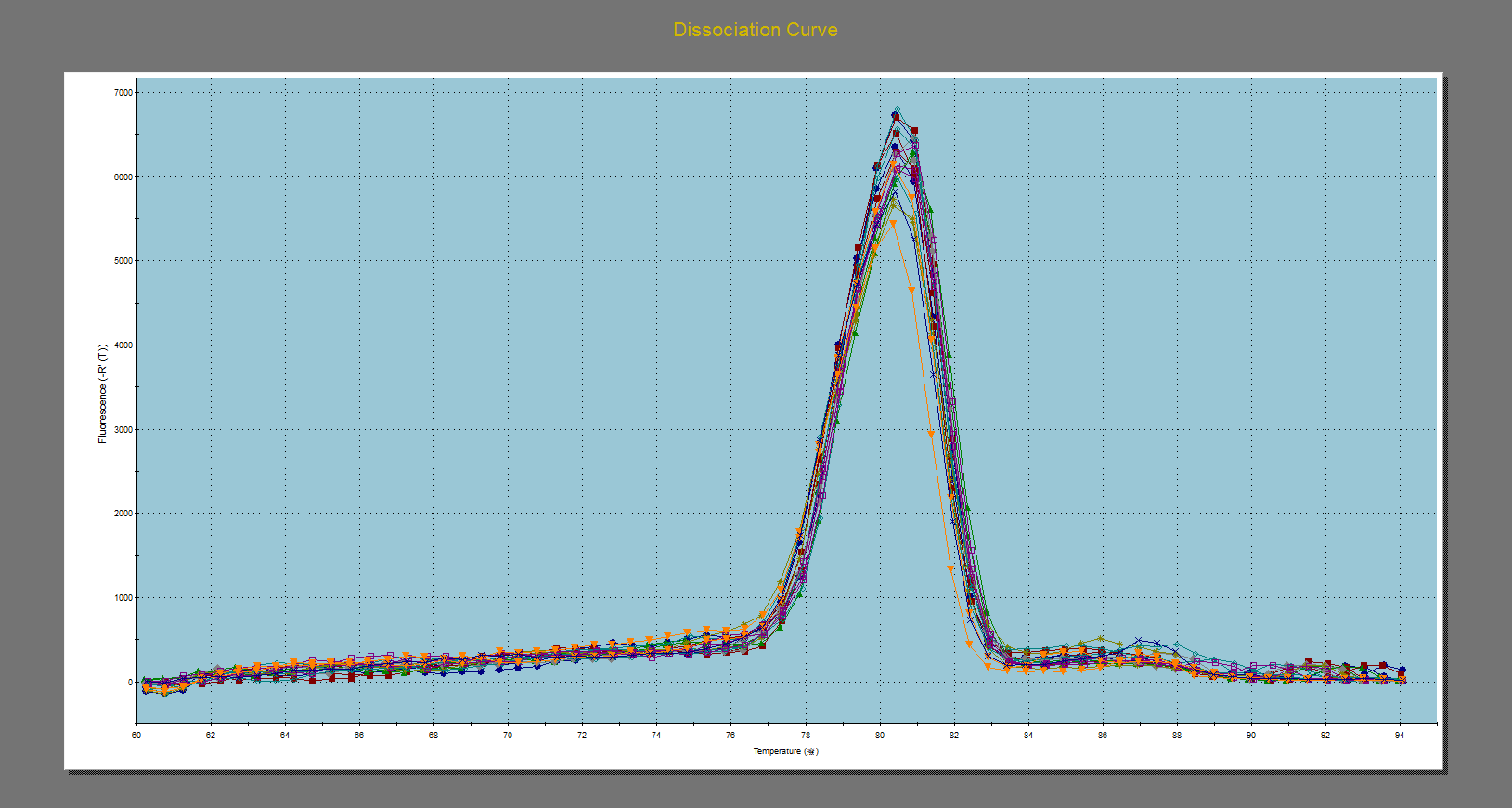

Supplement: File S2 — Contains 11 figures from Figure S14 to S24 representing the melting curves of qPCR products of the 10 candidate reference genes ( ACT, CYCL, DNAJ, EF1, EIF4A2, H2A, L2, TIP41, TUA and UBQ ) and one validation gene rbcL , respectively. (ZIP) [file pone.0104124.s006.zip › File S2/Figure S14 ACT.bmp]

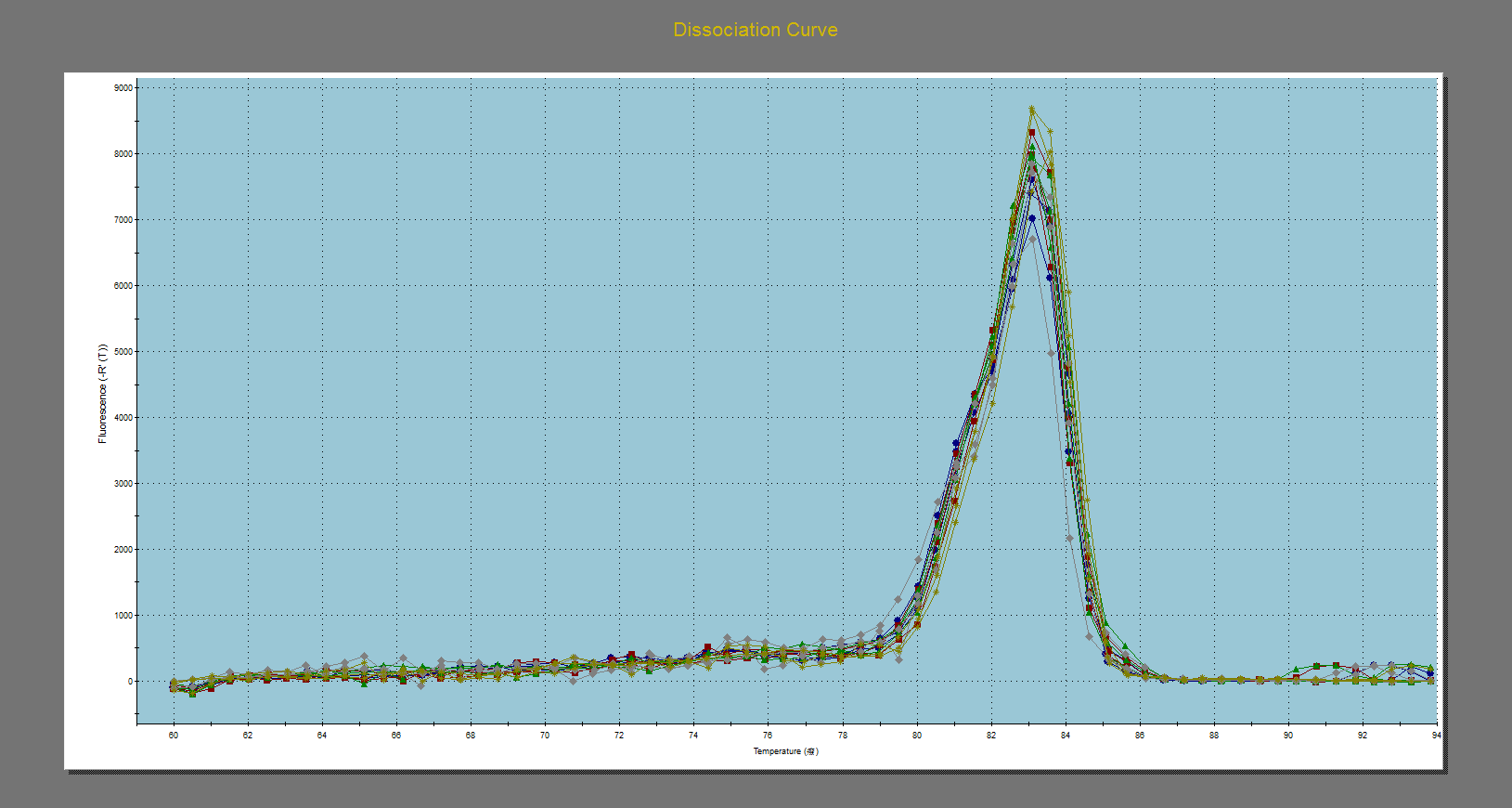

Supplement: File S2 — Contains 11 figures from Figure S14 to S24 representing the melting curves of qPCR products of the 10 candidate reference genes ( ACT, CYCL, DNAJ, EF1, EIF4A2, H2A, L2, TIP41, TUA and UBQ ) and one validation gene rbcL , respectively. (ZIP) [file pone.0104124.s006.zip › File S2/Figure S15 CYCL.bmp]

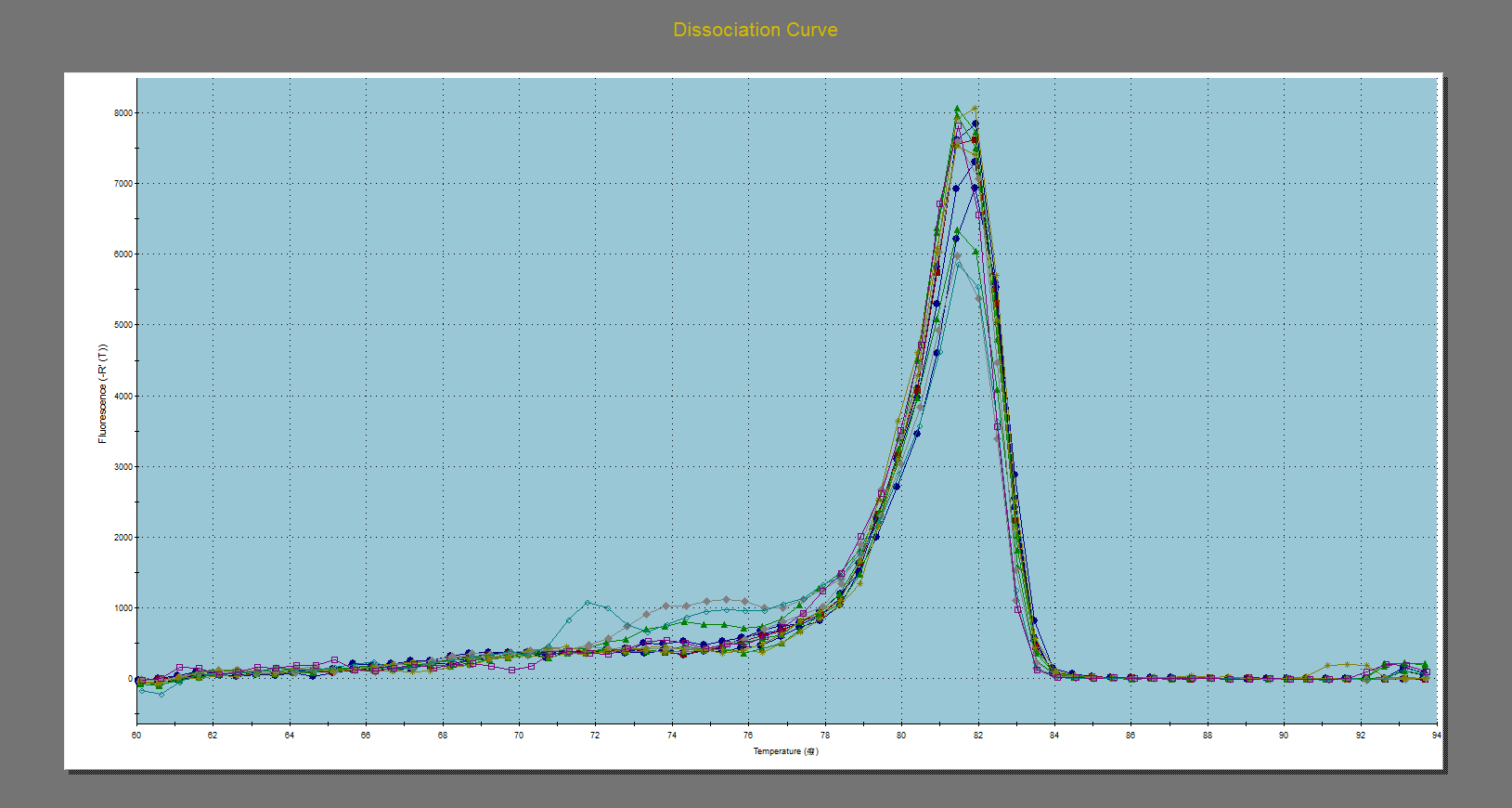

Supplement: File S2 — Contains 11 figures from Figure S14 to S24 representing the melting curves of qPCR products of the 10 candidate reference genes ( ACT, CYCL, DNAJ, EF1, EIF4A2, H2A, L2, TIP41, TUA and UBQ ) and one validation gene rbcL , respectively. (ZIP) [file pone.0104124.s006.zip › File S2/Figure S16 DNAJ.bmp]

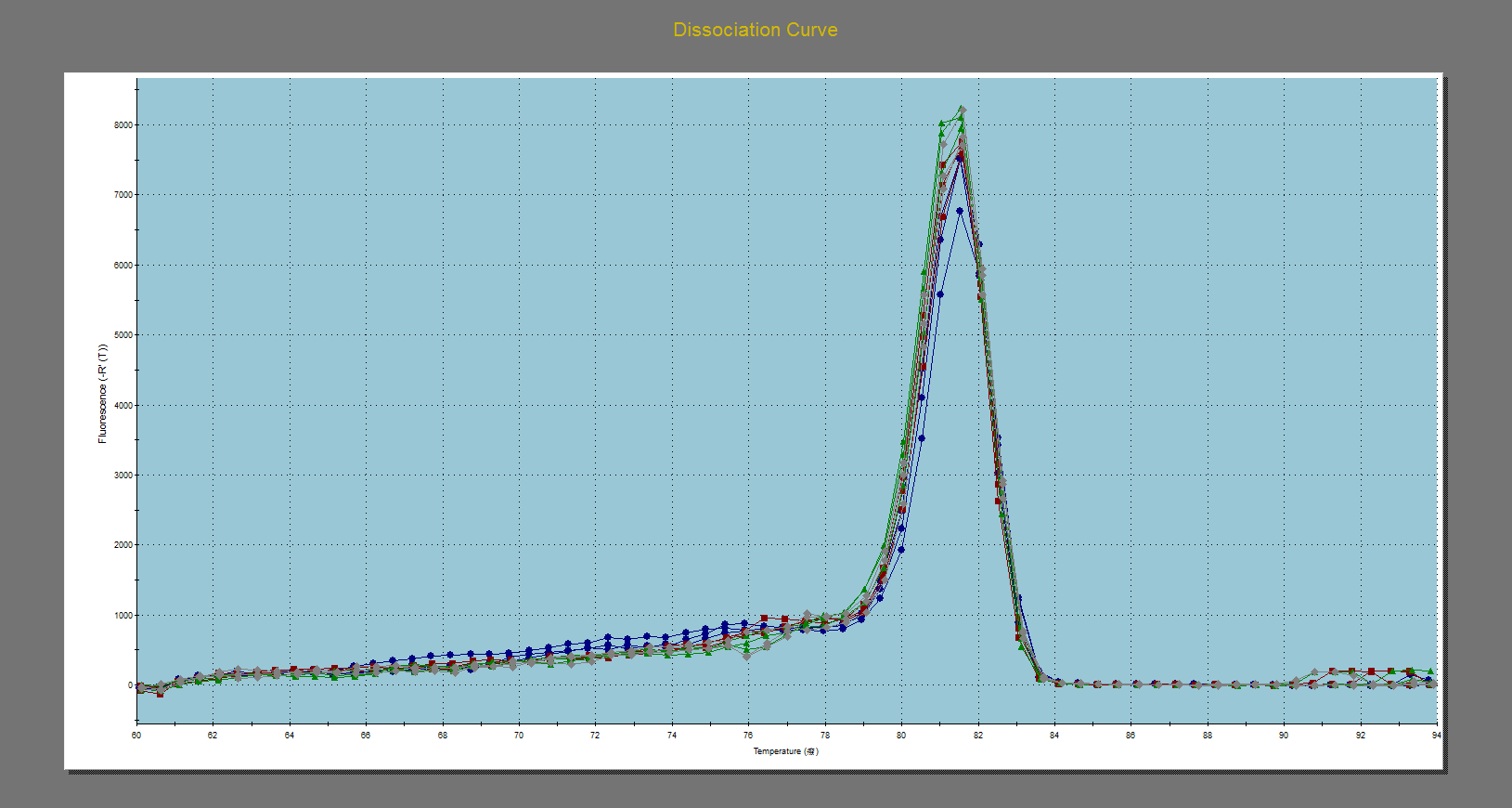

Supplement: File S2 — Contains 11 figures from Figure S14 to S24 representing the melting curves of qPCR products of the 10 candidate reference genes ( ACT, CYCL, DNAJ, EF1, EIF4A2, H2A, L2, TIP41, TUA and UBQ ) and one validation gene rbcL , respectively. (ZIP) [file pone.0104124.s006.zip › File S2/Figure S17 EF1a.bmp]

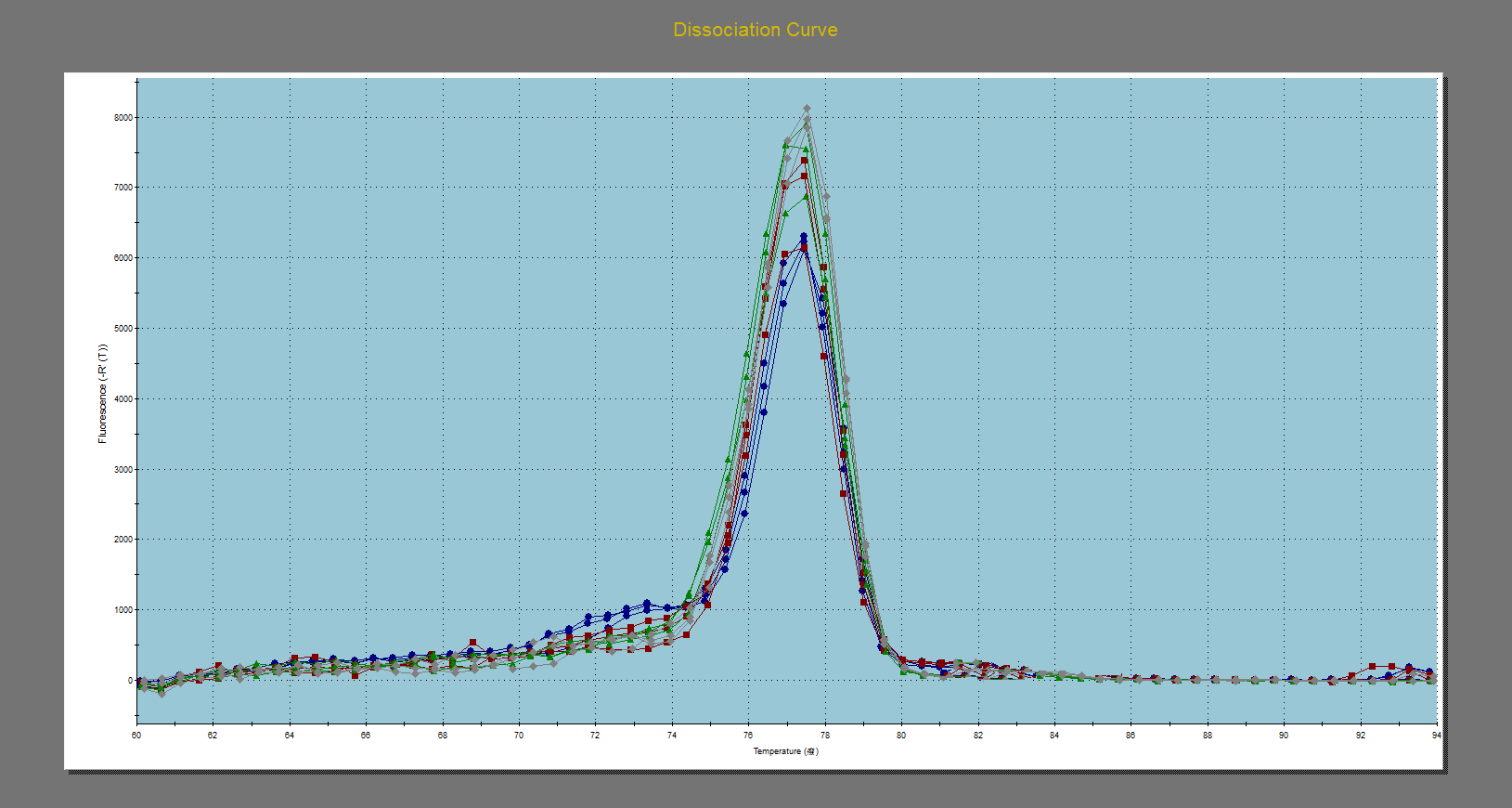

Supplement: File S2 — Contains 11 figures from Figure S14 to S24 representing the melting curves of qPCR products of the 10 candidate reference genes ( ACT, CYCL, DNAJ, EF1, EIF4A2, H2A, L2, TIP41, TUA and UBQ ) and one validation gene rbcL , respectively. (ZIP) [file pone.0104124.s006.zip › File S2/Figure S18 EIF4A2.bmp]

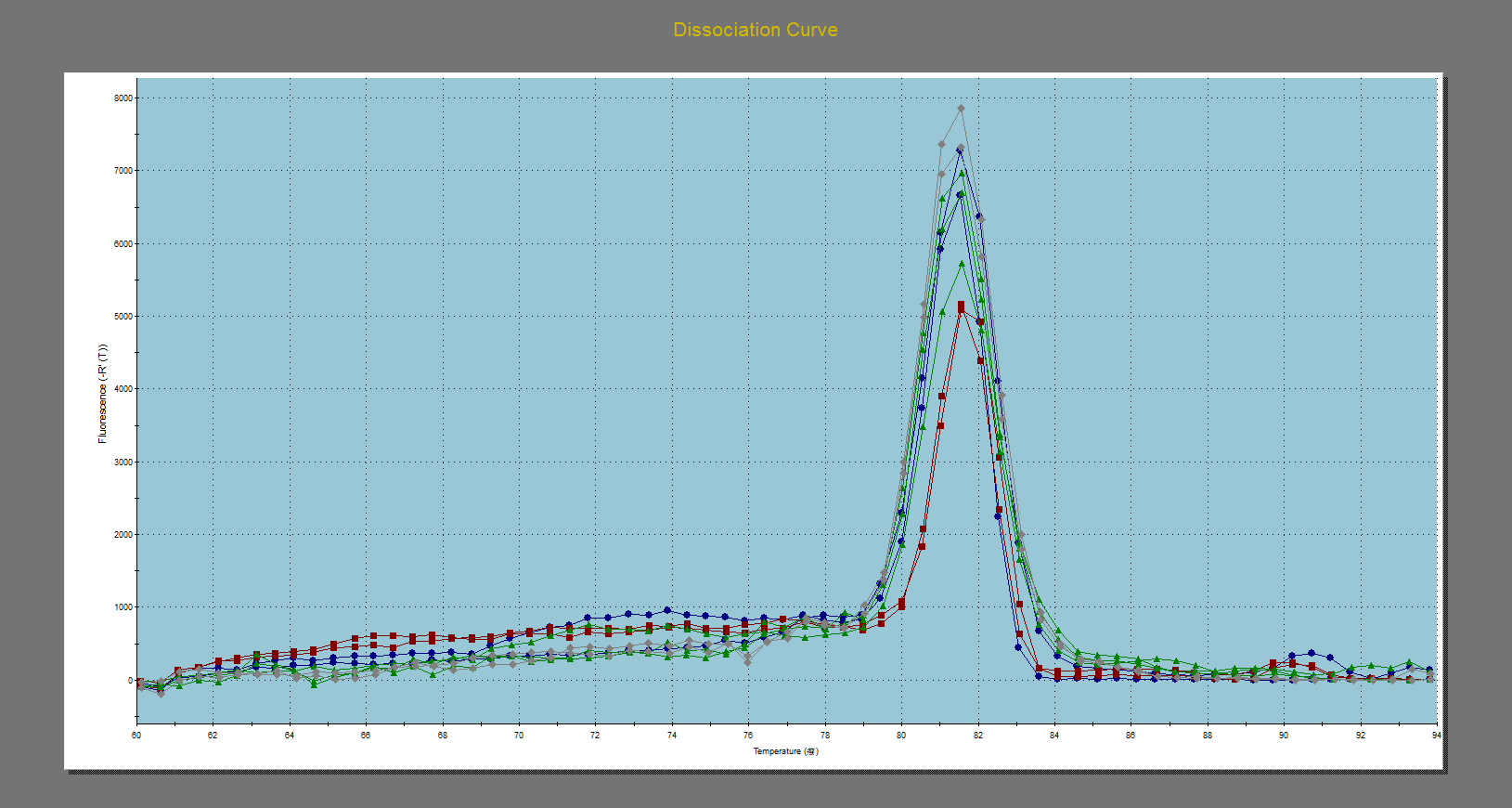

Supplement: File S2 — Contains 11 figures from Figure S14 to S24 representing the melting curves of qPCR products of the 10 candidate reference genes ( ACT, CYCL, DNAJ, EF1, EIF4A2, H2A, L2, TIP41, TUA and UBQ ) and one validation gene rbcL , respectively. (ZIP) [file pone.0104124.s006.zip › File S2/Figure S19 H2A.bmp]

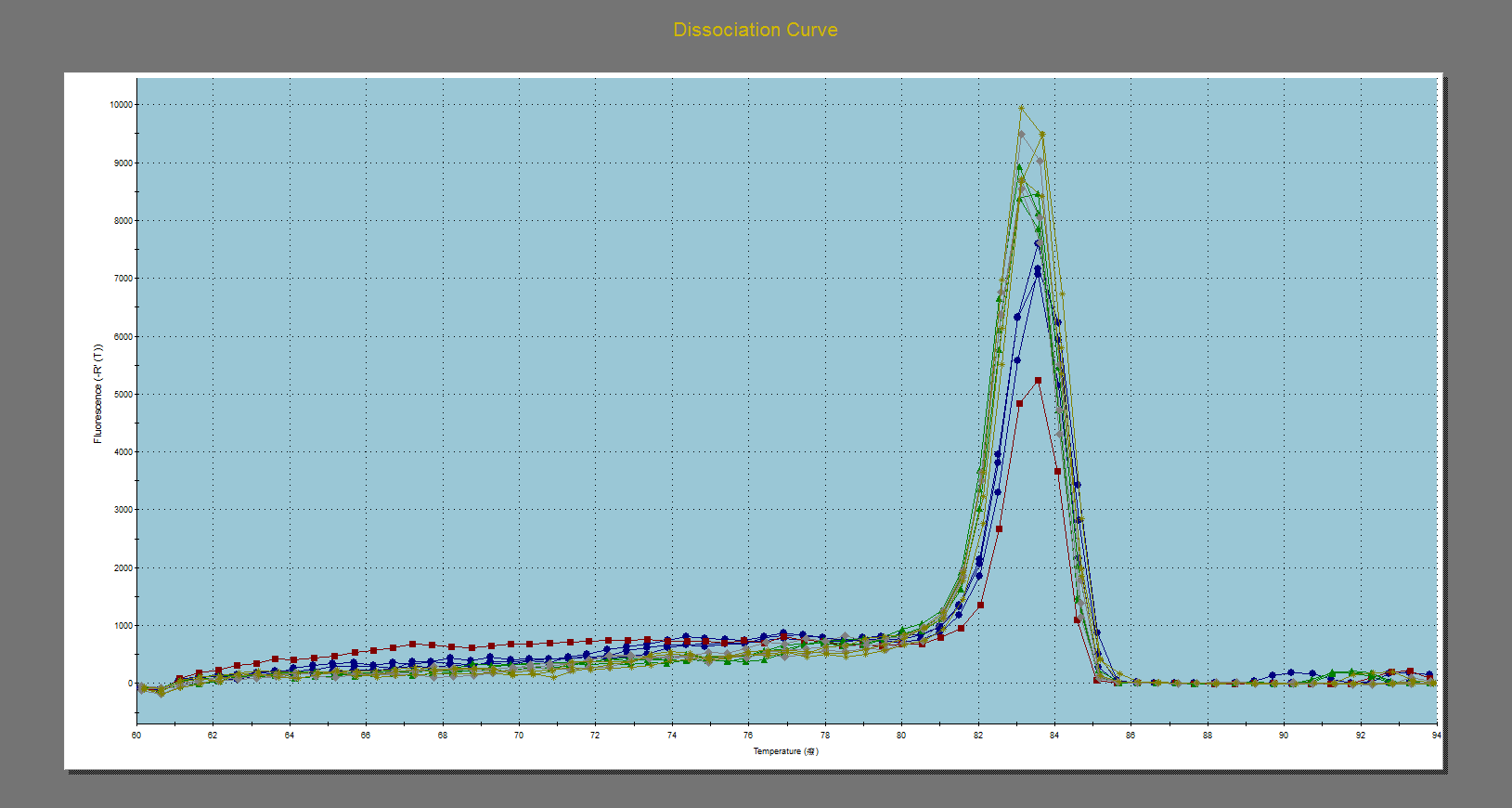

Supplement: File S2 — Contains 11 figures from Figure S14 to S24 representing the melting curves of qPCR products of the 10 candidate reference genes ( ACT, CYCL, DNAJ, EF1, EIF4A2, H2A, L2, TIP41, TUA and UBQ ) and one validation gene rbcL , respectively. (ZIP) [file pone.0104124.s006.zip › File S2/Figure S20 L2.bmp]

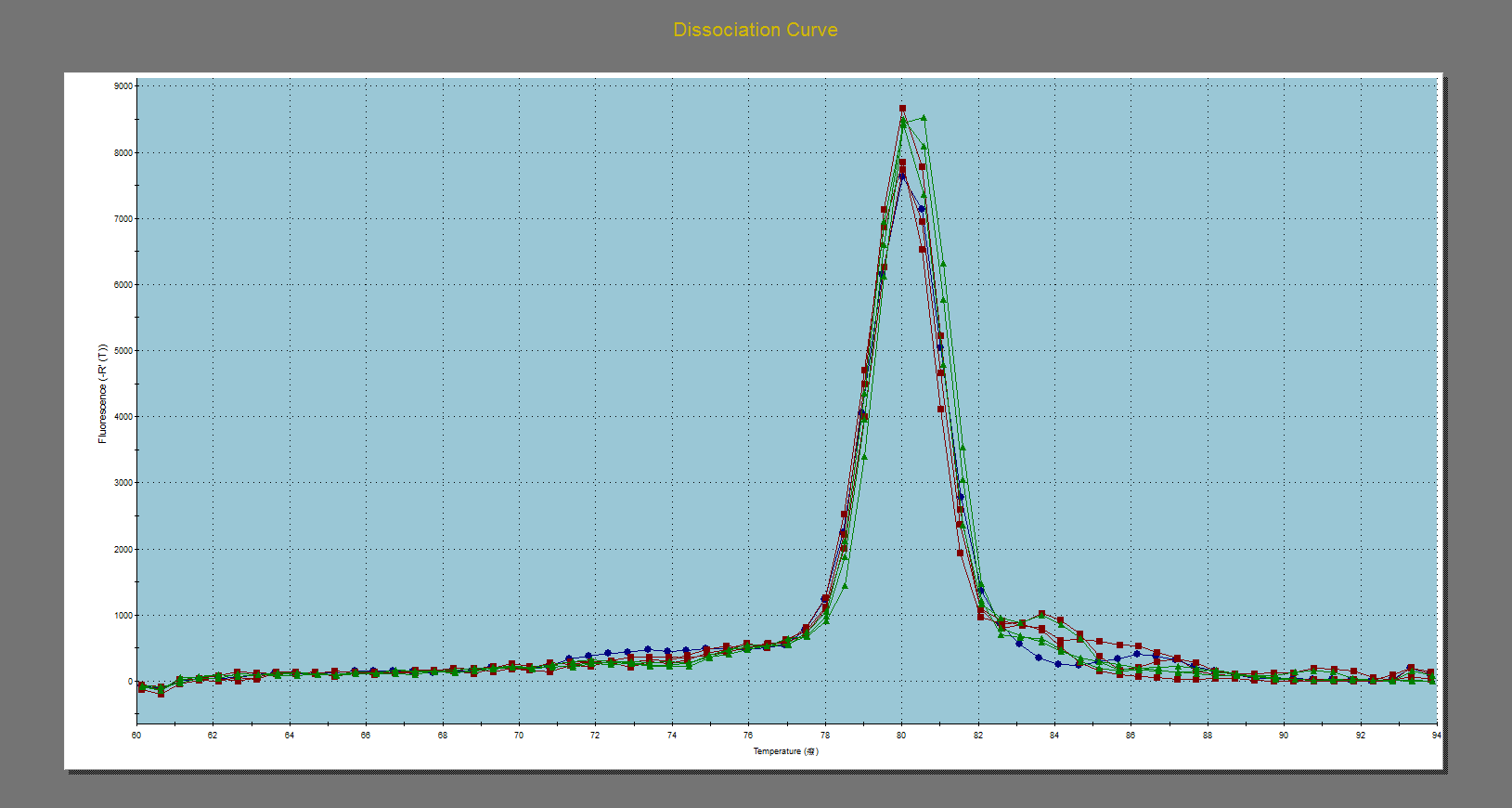

Supplement: File S2 — Contains 11 figures from Figure S14 to S24 representing the melting curves of qPCR products of the 10 candidate reference genes ( ACT, CYCL, DNAJ, EF1, EIF4A2, H2A, L2, TIP41, TUA and UBQ ) and one validation gene rbcL , respectively. (ZIP) [file pone.0104124.s006.zip › File S2/Figure S21 TIP41.bmp]

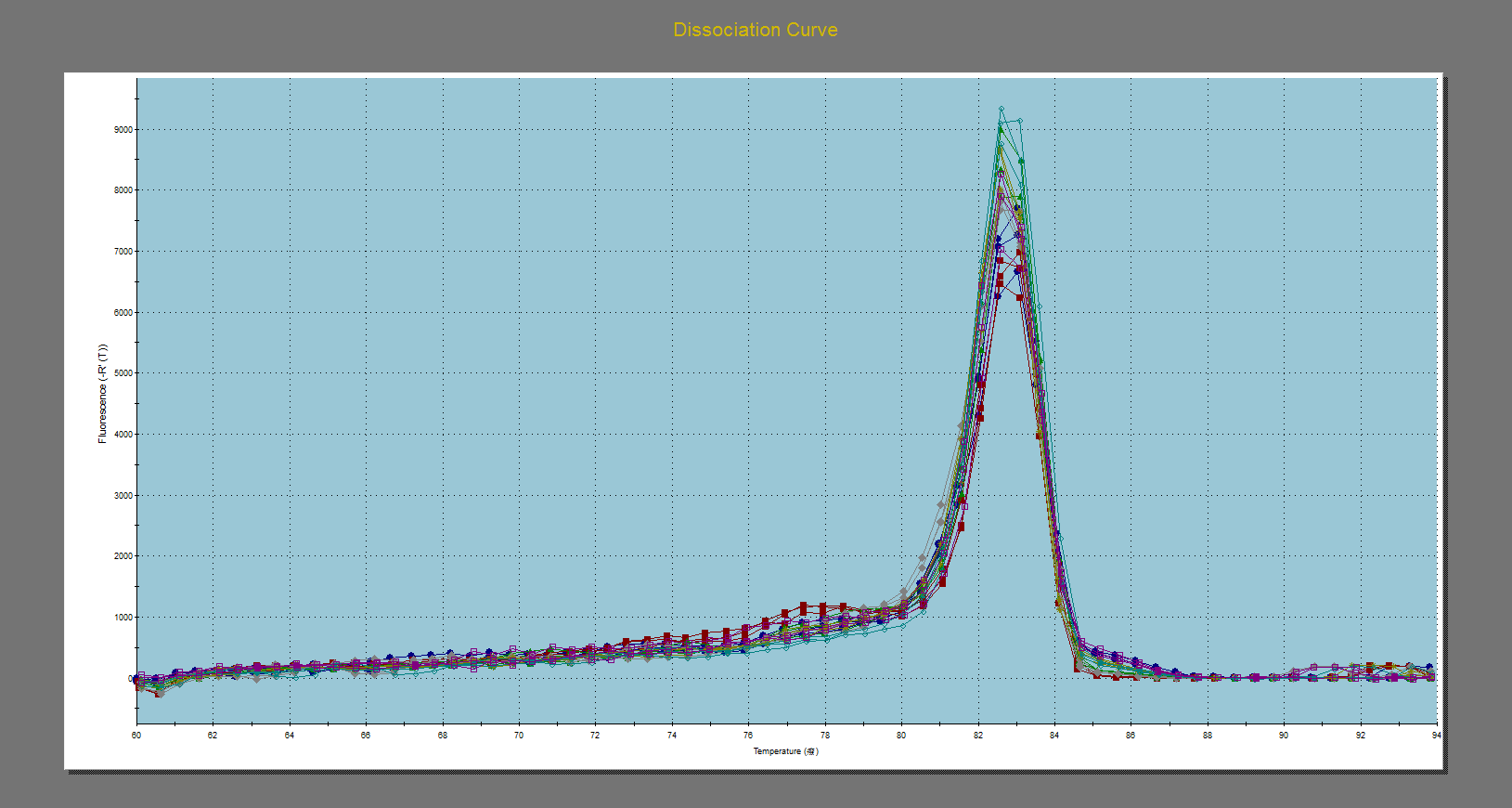

Supplement: File S2 — Contains 11 figures from Figure S14 to S24 representing the melting curves of qPCR products of the 10 candidate reference genes ( ACT, CYCL, DNAJ, EF1, EIF4A2, H2A, L2, TIP41, TUA and UBQ ) and one validation gene rbcL , respectively. (ZIP) [file pone.0104124.s006.zip › File S2/Figure S22 TUA.bmp]

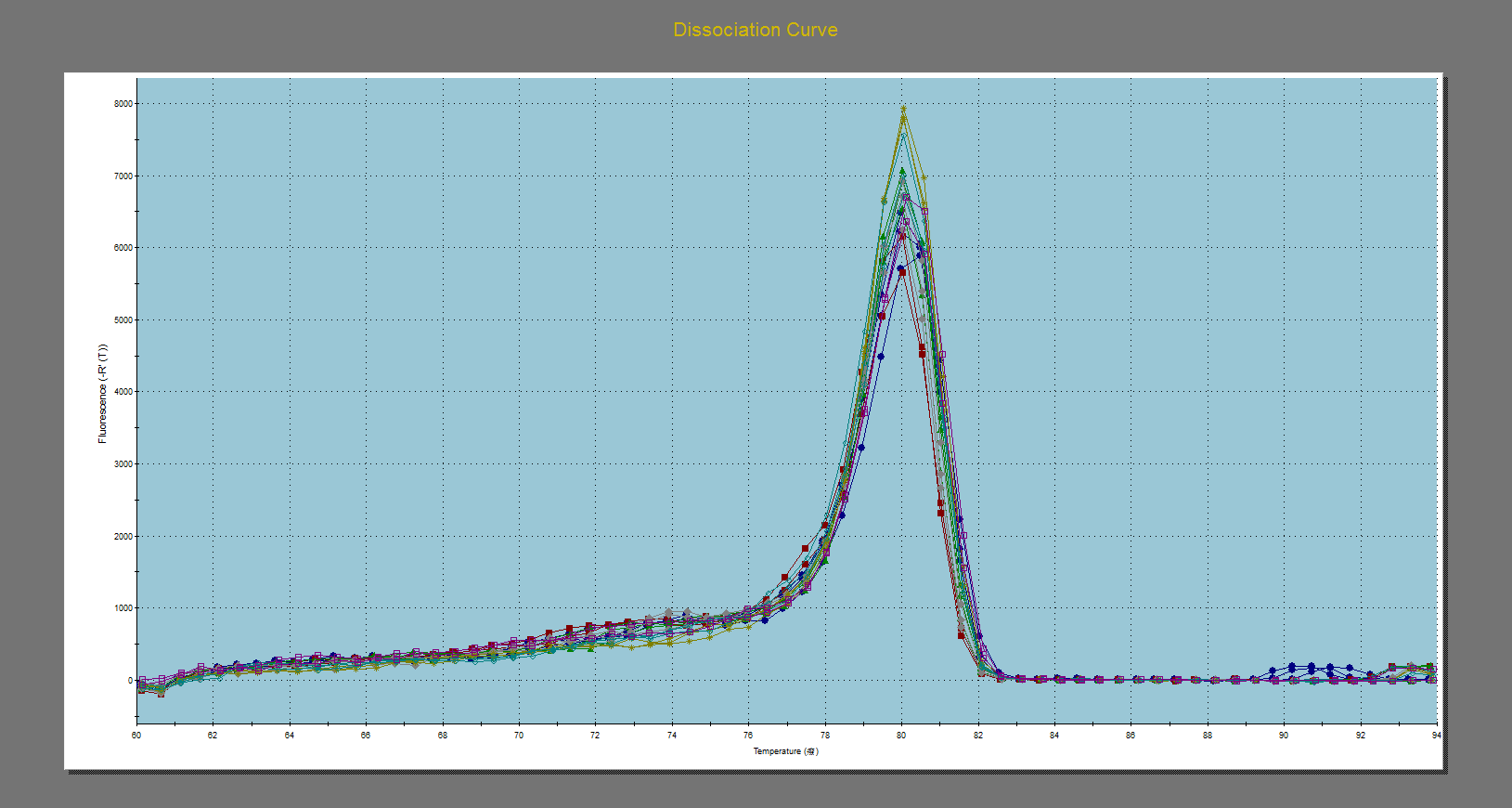

Supplement: File S2 — Contains 11 figures from Figure S14 to S24 representing the melting curves of qPCR products of the 10 candidate reference genes ( ACT, CYCL, DNAJ, EF1, EIF4A2, H2A, L2, TIP41, TUA and UBQ ) and one validation gene rbcL , respectively. (ZIP) [file pone.0104124.s006.zip › File S2/Figure S23 UBQ.bmp]

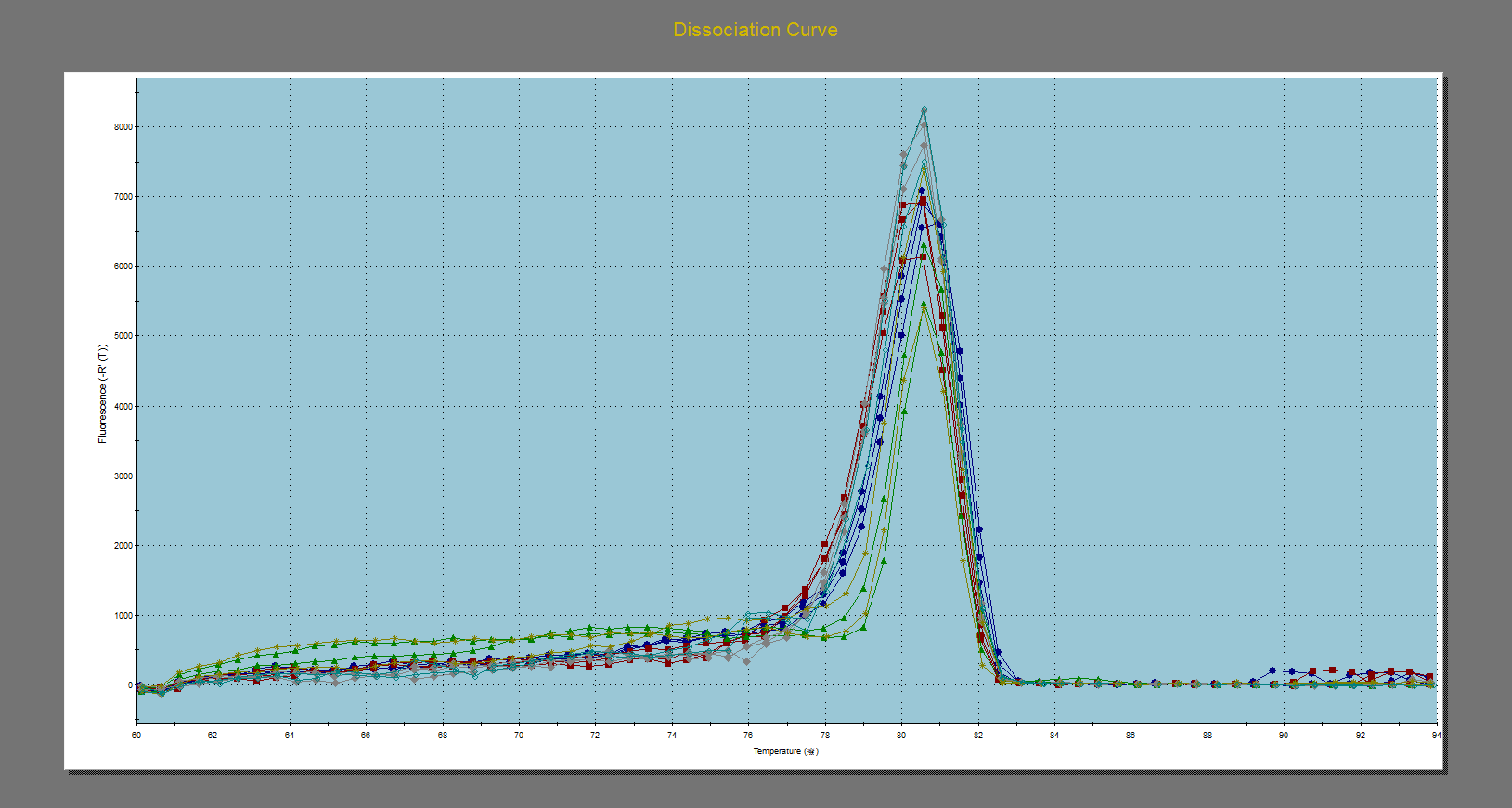

Supplement: File S2 — Contains 11 figures from Figure S14 to S24 representing the melting curves of qPCR products of the 10 candidate reference genes ( ACT, CYCL, DNAJ, EF1, EIF4A2, H2A, L2, TIP41, TUA and UBQ ) and one validation gene rbcL , respectively. (ZIP) [file pone.0104124.s006.zip › File S2/Figure S24 rbcL.bmp]
