# Supplementary material for: Reference Gene Selection for Quantitative Real-Time PCR Normalization in Reaumuria soongorica
Source: PLoS One. 2014 Aug 12;9(8):e104124. doi: 10.1371/journal.pone.0104124 (PMC4130609; doi:10.1371/journal.pone.0104124)
Supplement: Table S2 — Ranking of the best reference gene based on the all three algorithms. (DOC) [file pone.0104124.s004.doc]

| Table S1. The ranking of 10 reference genes by geNorm, NormFinder, and Bestkeeper. | | | | | | | | | | | |
| --- | --- | --- | --- | --- | --- | --- | --- | --- | --- | --- | --- |
| **Rank** | Software | 1 | 2 | 3 | 4 | 5 | 6 | 7 | 8 | 9 | 10 |
| **Total** | G | EIF4A2 | H2A | DnaJ | TIP41 | CYCl | ACT | UBQ | TUA | L2 | EF1α |
|  | N | EIF4A2 | ACT | H2A | DNAJ | CYCl | TUA | UBQ | TIP41 | L2 | EF1α |
|  | B | ACT | EIF4A2 | H2A | DNAJ | TUA | TIP41 | CYCl | EF1α | UBQ | L2 |
| **PEG** | G | CYCl | DnaJ | TIP41 | UBQ | H2A | ACT | EIF4A2 | CYCl | TUA | L2 |
|  | N | UBQ | CYCl | DNAJ | H2A | TIP41 | EF1α | ACT | EIF4A2 | TUA | L2 |
|  | B | ACT | H2A | UBQ | EF1α | L2 | TIP41 | CYCl | DNAJ | EIF4A2 | TUA |
| **Heat** | G | DnaJ | EF1a | UBQ | ACT | TUA | H2A | CYCL | EIF4A2 | L2 | TIP41 |
|  | N | EF1α | DNAJ | ACT | TUA | UBQ | CYCl | H2A | EIF4A2 | L2 | TIP41 |
|  | B | EF1α | DNAJ | ACT | H2A | CYCl | UBQ | TUA | EIF4A2 | L2 | TIP41 |
| **Dark** | G | EIF4A2 | H2A | DnaJ | TIP41 | CYCl | ACT | UBQ | TUA | L2 | EF1α |
|  | N | DNAJ | TIP41 | ACT | EIF4A2 | CYCl | UBQ | TUA | H2A | L2 | EF1α |
|  | B | ACT | DNAJ | TIP41 | TUA | EIF4A2 | CYCl | H2A | UBQ | EF1α | L2 |
| **Salt** | G | UBQ | TIP41 | ACT | CYCL | L2 | EF1α | EIF4A2 | H2A | TUA | DnaJ |
|  | N | UBQ | CYCl | TIP41 | TUA | L2 | EF1α | ACT | H2A | EIF4A2 | DNAJ |
|  | B | L2 | UBQ | EF1α | TIP41 | CYCl | ACT | EIF4A2 | H2A | TUA | DNAJ |

Notes: 1 represents the most stable gene and 10 represents the least stable gene; G: geNorm, N: NormFinder, B: Bestkeeper.
