# Supplementary material for: Reference Gene Selection for Quantitative Real-Time PCR Normalization in Reaumuria soongorica
Source: PLoS One. 2014 Aug 12;9(8):e104124. doi: 10.1371/journal.pone.0104124 (PMC4130609; doi:10.1371/journal.pone.0104124)
Supplement: File S3 — Alignment of the protein sequences of rbcL homologs. (PDF) [file pone.0104124.s007.pdf]

```
                                                  10        20        30        40        50        60        70        80
                                          ....|....|....|....|....|....|....|....|....|....|....|....|....|....|....|....|
YP_008575886.1_Eucalyptus umbr            MSPQTETKASVGFKAGVKDYKLTYYTPEYETKDTDILAAFRVTPQPGVPPEEAGAAVAAESSTGTWTTVWTDGLTSLDRY
YP_008576991.1_Eucalyptus spat            MSPQTETKASVGFKAGVKDYKLTYYTPDYETKDTDILAAFRVTPQPGVPPEEAGAAVAAESSTGTWTTVWTDGLTSLDRY
YP_008577671.1_Corymbia macula            MSPQTETKASVGFKAGVKDYKLTYYTPDYETKDTDILAAFRVTPQPGVPPEEAGAAVAAESSTGTWTTVWTDGLTSLDRY
YP_008577076.1_Eucalyptus torq            MSPQTETKASVGFKAGVKDYKLTYYTPDYETKDTDILAAFRVTPQPGVPPEEAGAAVAAESSTGTWTTVWTDGLTSLDRY
AFJ05166.1_Punica granatum                MSPQTETKASVGFKAGVKDYKLTYYTPEYETKDTDILAAFRVTPQPGVPPEEAGAAVAAESSTGTWTTVWTDGLTSLDRY
AAS83539.1_Toxicodendron orien            MSPQTETKASVGFKAGVKDYKLTYYTPEYETKDTDILAAFRVTPQPGVPPEEAGAAVAAESSTGTWTTVWTDGLTSLDRY
ABV65256.1_Quercus nigra                  MSPQTETKASVGFKAGVKDYKLTYYTPDYQTKDTDILAAFRVTPQPGVPPEEAGAAVAAESSTGTWTTVWTDGLTSLDRY
Unigene19142_R.soongorica                 MSPQTETKASVGFKAGVKDYKLTYYTPEYETKDTDILAAFRVTPQPGVPPEEAGAAVAAESSTGTWTTVWTDGLTSLDRY

                                                  90       100       110       120       130       140       150       160
                                          ....|....|....|....|....|....|....|....|....|....|....|....|....|....|....|....|
YP_008575886.1_Eucalyptus umbr            KGRCYHIEPVAGEENQYICYVAYPLDLFEEGSVTNMFTSIVGNVFGFKALRALRLEDLRIPTSYTKTFQGPPHGIQVERD
YP_008576991.1_Eucalyptus spat            KGRCYHIEPVAGEENQYICYVAYPLDLFEEGSVTNMFTSIVGNVFGFKALRALRLEDLRIPTSYTKTFQGPPHGIQVERD
YP_008577671.1_Corymbia macula            KGRCYHIEPVAGEENQYICYVAYPLDLFEEGSVTNMFTSIVGNVFGFKALRALRLEDLRIPTSYTKTFQGPPHGIQVERD
YP_008577076.1_Eucalyptus torq            KGRCYHIEPVAGEENQYICYVAYPLDLFEEGSVTNMFTSIVGNVFGFKALRALRLEDLRIPTSYTKTFQGPPHGIQVERD
AFJ05166.1_Punica granatum                KGRCYHIEPVAGEENQYICYVAYPLDLFEEGSVTNMFTSIVGNVFGFKALRALRLEDLRIPTAYTKTFQGPPHGIQVERD
AAS83539.1_Toxicodendron orien            KGRCYNIEPVAGEENQYICYVAYPLDLFEEGSVTNMFTSIVGNVFGFKALRALRLEDLRSPTAYTKTFQGPPHGIQVERD
ABV65256.1_Quercus nigra                  KGRCYHIEPVAGEENQFIAYVAYPLDLFEEGSVTNMFTSIVGNVFGFKALRALRLEDLRIPTSYSKTFQGPPHGIQVERD
Unigene19142_R.soongorica                 KGRCYHIEPVAGEENQYICYVAYPLDLFEEGSVTNMFTSIVGNVFGFKALRALRLEDLRIPTSYTKTFQGPPHGIQVERD

                                                 170       180       190       200       210       220       230       240
                                          ....|....|....|....|....|....|....|....|....|....|....|....|....|....|....|....|
YP_008575886.1_Eucalyptus umbr            KLNKYGRPLLGCTIKPKLGLSAKNYGRAVYECLRGGLDFTKDDENVNSQPFMRWRDRFLFCAEAIFKSQAETGEIKGHYL
YP_008576991.1_Eucalyptus spat            KLNKYGRPLLGCTIKPKLGLSAKNYGRAVYECLRGGLDFTKDDENVNSQPFMRWRDRFLFCAEAIFKSQAETGEIKGHYL
YP_008577671.1_Corymbia macula            KLNKYGRPLLGCTIKPKLGLSAKNYGRAVYECLRGGLDFTKDDENVNSQPFMRWRDRFLFCAEAIFKSQAETGEIKGHYL
YP_008577076.1_Eucalyptus torq            KLNKYGRPLLGCTIKPKLGLSAKNYGRAVYECLRGGLDFTKDDENVNSQPFMRWRDRFLFCAEAIFKSQAETGEIKGHYL
AFJ05166.1_Punica granatum                KLNKYGRPLLGCTIKPKLGLSAKNYGRAVYECLRGGLDFTKDDENVNSQPFMRWRDRFLFCAEALYKAQAETGEIKGHYL
AAS83539.1_Toxicodendron orien            KLNKYGRPLLGCTIKPKLGLSAKNYGRAVYECLRGGLDFTKDDENVNSQPFMRWRDRFLFCAEALYKAQAETGEIKGHYL
ABV65256.1_Quercus nigra                  KLNKYGRPLLGCTIKPKLGLSAKNYGRAVYECLRGGLDFTKDDENVNSQPFMRWRDRFVFCAEAIYKAQAETGEIKGHYL
Unigene19142_R.soongorica                 KLNKYGRPLLGCTIKPKLGLSAKNYGRAVYECLRGGLDFTKDDENVNSQPFMRWRDRFLFCAEAIFKAQGETGEIKGHYL

                                                 250       260       270       280       290       300       310       320
                                          ....|....|....|....|....|....|....|....|....|....|....|....|....|....|....|....|
YP_008575886.1_Eucalyptus umbr            NATAGTCEEMIKRAVFARELGVPIVMHDYLTGGFTANTSLAHYCRDNGLLLHIHRAMHAVIDRQKNHGIHFRVLAKALRM
YP_008576991.1_Eucalyptus spat            NATAGTCEEMIKRAVFARELGVPIVMHDYLTGGFTANTSLAHYCRDNGLLLHIHRAMHAVIDRQKNHGIHFRVLAKALRM
YP_008577671.1_Corymbia macula            NATAGTCEEMIKRAVFARELGVPIVMHDYLTGGFTANTSLAHYCRDNGLLLHIHRAMHAVIDRQKNHGIHFRVLAKALRM
YP_008577076.1_Eucalyptus torq            NATAGTCEEMIKRAVFARELGAPIVMHDYLTGGFTANTSLAHYCRDNGLLLHIHRAMHAVIDRQKNHGIHFRVLAKALRM
AFJ05166.1_Punica granatum                NATAGTCEEMIKRAVFARELGAPIVMHDYLTGGFTANTSLAHYCRDNGLLLHIHRAMHAVIDRQKNHGMHFSVLAKALRM
AAS83539.1_Toxicodendron orien            NATAGTCEEMLKRAVFARELGVPIVMHDYLTGGFTANTSLAHYCRDNGLLLHIHRAMHAVIDRQKNHGMHFRVLAKALRM
ABV65256.1_Quercus nigra                  NATAGTCEEMIKRAVFARELGVPIVMHDYLTGGFTANTSLAHYCRDNGLLLHIHRAMHAVIDRQKNHGIHFRVLAKALRM
Unigene19142_R.soongorica                 NATAGTCEEMIKRAVFARELGVPIVMHDYLTGGFTANTSLAHYCRDNGLLLHIHRAMHAVIDRQKNHGIHFRVLAKALRM

                                                 330       340       350       360       370       380       390       400
                                          ....|....|....|....|....|....|....|....|....|....|....|....|....|....|....|....|
YP_008575886.1_Eucalyptus umbr            SGGDHIHSGTVVGKLEGERDITLGFVDLLRDDFIEKDRSRGIYFTQDWVSLPGVLPVASGGIHVWHMPALTEIFGDDSVL
YP_008576991.1_Eucalyptus spat            SGGDHIHAGTVVGKLEGERDITLGFVDLLRDDFIEKDRSRGIYFTQDWVSLPGVLPVASGGIHVWHMPALTEIFGDDSVL
YP_008577671.1_Corymbia macula            SGGDHIHSGTVVGKLEGERDITLGFVDLLRDDFIEKDRSRGIYFTQDWVSLPGVLPVASGGIHVWHMPALTEIFGDDSVL
YP_008577076.1_Eucalyptus torq            SGGDHIHAGTVVGKLEGERDITLGFVDLLRDDFIEKDRSRGIYFTQDWVSLPGVLPVASGGIHVWHMPALTEIFGDDSVL
AFJ05166.1_Punica granatum                SGGDHIHAGTVVGKLEGERDITLGFVDLLRDDFIEKDRSRGIYFTQDWVSLPGVLPVASGGIHVWHMPALTEIFGDDSVL
AAS83539.1_Toxicodendron orien            SGGDHIHAGTVVGKLEGERDITLGFVDLLRDDFIEKDRSRGIYFTQDWVSLPGVLPVASGGIHVWHMPALTEIFGDDSVL
ABV65256.1_Quercus nigra                  SGGDHIHAGTVVGKLEGEREITLGFVDLLRDDYIEKDRSRGIYFTQDWVSLPGVLPVASGGIHVWHMPALTEIFGDDSVL
Unigene19142_R.soongorica                 SGGDHIHAGTVVGKLEGEREITLGFVDLLRDDYIEKDRSRGIYFTQPWVSLPGVLPVASGGIHVWHMPALTEIFGDDSVL

                                                 410       420       430       440       450       460       470
                                          ....|....|....|....|....|....|....|....|....|....|....|....|....|....|
YP_008575886.1_Eucalyptus umbr            QFGGGTLGHPWGNAPGAVANRVALEACVQARNEGRDLAREGNEIIREASKWSPELAAACEVWKEIKFEFEAMDTL
YP_008576991.1_Eucalyptus spat            QFGGGTLGHPWGNAPGAVANRVALEACVQARNEGRDLAREGNEIIREASKWSPELAAACEVWKEIKFEFEAMDTL
YP_008577671.1_Corymbia macula            QFGGGTLGHPWGNAPGAVANRVALEACVQARNEGRDLAREGNEIIREASKWSPELAAACEVWKEIKFEFEAMDTL
YP_008577076.1_Eucalyptus torq            QFGGGTLGHPWGNAPGAVANRVALEACVQARNEGRDLAREGNEIIREASKWSPELAAACEVWKEIKFEFEAMDTL
AFJ05166.1_Punica granatum                QFGGGTLGHPWGNAPGAVANRVALEACVQARNEGRDLAREGNEIIREASKWSPELAAACEVWKEIKFEFEAMDTL
AAS83539.1_Toxicodendron orien            QFGGGTLGHPWGNAPGAVANRVALEACVQARNEGRDLAREGNEIIREASKWSPELAAACEVWKEIKFEFEAMDTL
ABV65256.1_Quercus nigra                  QFGGGTLGHPWGNAPGAVANRVALEACVQARNEGRDLAREGNEIIREAAKWSPELAAACEVWKEIKFEFPAMDTL
Unigene19142_R.soongorica                 QFGGGTLGHPWGNAPGAVANRVALEACVQARNEGRDLAREGNEIIREACKWSPELAAACEVWKEIKFEFEAMDTL
```
